# The Surface of Nanoparticle Silicon as Studied by Solid-State NMR

**DOI:** 10.3390/ma6010018

**Published:** 2012-12-20

**Authors:** Rebecca A. Faulkner, Joseph A. DiVerdi, Yuan Yang, Takeshi Kobayashi, Gary E. Maciel

**Affiliations:** Department of Chemistry, Colorado State University, Fort Collins, CO 80523, USA; E-Mails: rebecca.fai;lmer32@gmail.com (R.A.F.); joseph.diverdi@colostate.edu (J.A.D.); yuanyang@mines.edu (Y.Y.); tkobayashi@ameslab.gov (T.K.)

**Keywords:** silicon, nanoparticles, ^29^Si NMR, solid-state NMR, surface characterization

## Abstract

The surface structure and adjacent interior of commercially available silicon nanopowder (*np*-Si) was studied using multinuclear, solid-state NMR spectroscopy. The results are consistent with an overall picture in which the bulk of the *np*-Si *interior* consists of highly ordered (“crystalline”) silicon atoms, each bound tetrahedrally to four other silicon atoms. From a combination of ^1^H, ^29^Si and ^2^H magic-angle-spinning (MAS) NMR results and quantum mechanical ^29^Si chemical shift calculations, silicon atoms on the *surface* of “as-received” *np*-Si were found to exist in a variety of chemical structures, with apparent populations in the order (a) (*Si*–O–)_3_**Si**–H > (b) (*Si*–O–)_3_**Si**OH > (c) (HO–)*_n_***Si**(*Si*)*_m_*(–O*Si*)_4−*m*−*n*_ ≈ (d) (*Si*–O–)_2_**Si**(H)OH > (e) (*Si*–O–)_2_**Si**(–OH)_2_ > (f) (*Si*–O–)_4_**Si**, where **Si** stands for a surface silicon atom and *Si* represents another silicon atom that is attached to **Si** by either a **Si**–*Si* bond or a **Si**–O–*Si* linkage. The relative populations of each of these structures can be modified by chemical treatment, including with O_2_ gas at elevated temperature. A deliberately oxidized sample displays an increased population of (*Si*–O–)_3_**Si**–H, as well as (*Si*–O–)_3_**Si**OH sites. Considerable heterogeneity of some surface structures was observed. A combination of ^1^H and ^2^H MAS experiments provide evidence for a substantial population of silanol (**Si**–OH) moieties, some of which are not readily H-exchangeable, along with the dominant **Si**–H sites, on the surface of “as-received” *np*-Si; the silanol moieties are enhanced by deliberate oxidation. An extension of the DEPTH background suppression method is also demonstrated that permits measurement of the T_2_ relaxation parameter simultaneously with background suppression.

## 1. Introduction

Silicon particles, like those of other elements, often exhibit novel and unusual properties as their physical dimensions approach the nanometer scale [1]. Optical, physical and electronic properties of nanoparticle silicon (*np*-Si) can be very different from those of bulk silicon and are interesting from both a fundamental perspective and a practical viewpoint [1,2]. *np*-Si has been found to be biocompatible, allowing for its use in biological tracers [3], sensors [2], and carriers of pharmaceuticals [3] or other probes. Nanostructure silicon has shown potential as a chemical hydride (a compound that can take up and release molecular hydrogen without passing through the gas phase) [4]. The chemical nature and structural organization of silicon’s surface atoms play a crucial role in the behavior and characteristics of *np*-Si; and, although it has been subjected to considerable scrutiny [5], much remains to be understood about the surface structure and reactivity.

Silicon exists in several important and distinct allotropes. Crystalline silicon (*c*-Si) (available as large single crystals with high atomic purity, from which correspondingly large and thin wafers are derived) consists of silicon atoms in tetrahedral environments with each atom bonded to four other silicon atoms. This arrangement persists throughout the bulk solid and is discontinued at the surface, beyond which there are no additional silicon atoms (*i.e.*, no way to provide a total of four Si–Si bonds). If no effort is made to address this valence insufficiency, then there will be a number of “dangling bonds” (equivalently, trapped free radicals) associated with these surface silicon atoms [6]. Hydrogen [7], oxygen [8], halogens [9] or organic ligands [10] can be used to satisfy these bonding sites in a process commonly known as “passivation” [11], “capping” [12] or “termination” [9,10].

Porous silicon (*p*-Si) is obtained by anodic etching of crystalline silicon, in aqueous HF, resulting in a film of porous silicon on the surface of the wafer [13]. This highly porous film exhibits a large surface area and high spatial inhomogeneity, with both silicon structures and pores ranging in size from meso- to nano-scale [13,14,15,16,17]. The crystallinity of the source silicon is believed to be retained in porous silicon [13,16,17].

Another allotrope is amorphous silicon (*a*-Si), produced by condensing silicon vapor generated by RF-plasma-sputtering of a silicon wafer on a condensing target that is maintained in an ultra high vacuum (UHV) or an inert atmosphere, for example argon [18,19]. This allotrope is a non-crystalline solid that displays a high concentration of dangling bonds [18]. Hydrogenated amorphous silicon (*a*-Si:H) can be synthesized using the same method by including hydrogen or silane gas in the blanketing atmosphere [18,19]. Hydrogenated amorphous silicon has a lower concentration of dangling bonds than in amorphous silicon, *a*-Si [19].

Silicon nanoparticles (*np*-Si) can be prepared via solid-state and mixed-phase methods. Depending on the method used, the preparation can result in various types of surface termination [1,12].

The chemical structures present on the surface of the various forms of silicon vary widely and can be modified by various treatments. A silicon surface that is terminated with any variety of chemical functionalities can be manipulated in much the same fashion as are other liquid- or solid-state silicon species [20]. Reaction of the native silicon surface with O_2_ at high temperature is believed to result in the formation of a silicon oxide layer that may extend with significant depth into several layers of silicon atoms [8,21,22,23]. This oxidation reaction and its product are of extreme interest and importance to silicon device technologies.

The chemical and physical information that is currently available on the various elemental silicon systems has been obtained primarily through two significantly disparate approaches: (1) The study of ultra-low surface-area materials (~10^−4^ m^2^ g^−1^), using ultra-high vacuum (UHV) instrumentation and the techniques of “surface science”, that is, infra-red (IR) spectroscopy [24], scanning tunneling microscopy (STM) [25], atomic force microscopy (ATM) [26], transmission electron microscopy (TEM) [4,27], high-resolution electron energy loss spectroscopy (HREELS) [28] or X-ray photoelectron spectroscopy (XPS) [27], *etc.*, and (2) the study of high-surface-area materials (~10^2^ m^2^ g^−1^) by solid-state nuclear magnetic resonance (NMR) [13,14,15,16,17,18,19]. The surface of single crystal silicon has been extensively examined by the former approach but has been inaccessible to NMR, because there is an insufficient number of nuclei at the surface to overcome NMR’s inherently low sensitivity. All the other silicon allotropes have been studied by solid-state NMR, because their higher surface areas provide a sufficient number of nuclei for achieving satisfactory signal-to-noise ratios.

In the work described here, a commercial preparation of *np*-Si, termed “silicon nanopowder”, serves as a suitable material for the detailed solid-state NMR examination of the types and relative populations of structures present on the surface. The primary goal is to understand the nature of the nanoparticle surface in terms of its fundamental chemical properties. Our approach involves a combination of chemical treatments and solid-state NMR measurements. *np*-Si offers a high enough surface area to satisfy the requirement for a sufficient number of nuclei in the sample for NMR analysis.

## 2. Experimental

### 2.1. Samples

*Materials.* Silicon Nanopowder (98+%, #633097, Aldrich, batch 04421BH), designated here *np*-Si, with a reported average particle size of 50 nm, was used as described in the sections on sample preparation. ^13^C NMR measurements (see *Supporting Materials*) show that the as-received material contains less than 1% carbon by weight. ^2^H_2_O (99.9 atom % as ^2^H, Cambridge Isotope Labs.), tris(trimethylsilyl)silane (TTMSS, Fluka) and polydimethylsiloxane (PDMS, 2.5 MDa MW, Petrarch Systems, Inc. Bristol, PA) were used as received. All other materials used were reagent grade. O_2_ gas was used as received from Airgas (99+%, UN1072).

*Evacuated np-Si.* All samples, unless otherwise noted, were evacuated for 15 h at 5 × 10^−3^ Torr and at 150, 300, or 500 °C, as specified.

*Pentane-Treated np-Si.* The original nanopowder sample was suspended in pentane at a ratio of 25 mL pentane per gram, with swirling at room temperature for a few minutes, after which the mixture was centrifuged and the clear and colorless supernatant liquid was decanted. This process was repeated two more times and the resultant solid was evacuated for 15 h at 5 × 10^−3^ Torr and at 150, 300, or 500 °C.

*^2^H_2_O-Treated np-Si*. The original nanopowder sample was suspended in ^2^H_2_O at a ratio of 25 mL ^2^H_2_O per gram with occasional swirling at room temperature for 1 h, after which the mixture was centrifuged and the cloudy and yellow supernatant liquid was decanted. This process was repeated two more times and the resultant solid was evacuated for 15 h at 5 × 10^−3^ Torr and at 150 °C.

*^1^H_2_O-Treated np-Si.* ^1^H_2_O-treated *np*-Si samples were prepared by suspending the original sample in ^1^H_2_O at a ratio of 25 mL ^1^H_2_O per gram with occasional swirling at room temperature for three hours, after which the mixture was centrifuged and the cloudy and yellow supernatant liquid decanted. The resultant solid was evacuated for 15 h at 5 × 10^−3^ Torr and at 150 °C.

*CH_3_OH-Treated np-Si.* CH_3_OH-treated *np*-Si samples were prepared by suspending the original sample in CH_3_OH at a ratio of 25 mL CH_3_OH per gram with occasional swirling at room temperature for three hours, after which the mixture was centrifuged and the cloudy and yellow supernatant liquid decanted. The resultant solid was evacuated for 15 h at 5 × 10^−3^ Torr and at 150 °C.

*Oxidized np-Si.* O_2_ gas at atmospheric pressure was passed for one hour over the original sample contained in a ceramic boat placed in a furnace at 500 °C. After cooling to room temperature (under flowing O_2_) the solid was either used directly or treated as in the ^1^H_2_O-treated *np*-Si samples, including the final 150 °C evacuation.

*Sample Handling*. As-received silicon nanopowder and prior-to-evacuation *np*-Si samples were stored and handled in a common laboratory atmosphere with no action to prevent exposure to atmospheric oxygen or water, except as noted. After evacuation, all *np*-Si samples were handled exclusively in a dry N_2_ atmosphere. Samples for spectroscopy were contained in PENCIL-II (Chemagnetics, Fort Collins, CO), zirconia MAS rotors with standard plastic-to-ceramic closures. Unless specified to the contrary below in specific cases, *all* samples were evacuated at 150 °C before NMR measurements.

### 2.2. Techniques and Measurements

*Surface Area.* BET surface area measurements were made using a Quantasorb MS-6 system (Quantachrome, Boynton Beach, FL).

*X-Ray Powder Diffractometry*. X-ray diffraction (XRD) powder pattern measurements were performed using a calibrated Scintag X2 Advanced Diffraction System (Themo Optek, Cupertino, CA) equipped with Cu K*α* radiation (*λ* = 0.154 nM) and a Peltier-cooled detector. Diffraction patterns were recorded in the 2*θ* range of 5° to 90° with a step size of 0.02° at a rate of 1° min^−1^. Diffrac^plus^ EVA software (Bruker AXS, Madison, WI) with the PDF-2 database (International Centre for Diffraction Data, Newtown Square, PA) was used for background correction and peak assignment. XRD samples were prepared by dusting silicon nanopowder onto glass substrates.

*DP ^29^Si NMR.* Direct-polarization (DP) solid-state ^29^Si NMR spectra were obtained using a CMX-II spectrometer (Chemagnetics, Inc. Fort Collins, CO) operating at 4.7 T (39.8 MHz for ^29^Si and 199.9 MHz for ^1^H) and employing a commercial (Chemagnetics) MAS probe with a 7.0 mm MAS system with a spinning speed of 5.5–5.8 kHz. An initial state of transverse ^29^Si magnetization was created using a single ~30° ^29^Si pulse (2.0 µs). High power ^1^H decoupling (46 kHz) was used. Spectra were obtained by Fourier transformation of complex data after apodization with 40 Hz of Lorentzian line broadening. The recovery time was 30 s.

*CP ^29^Si NMR.* Cross-polarization solid-state ^29^Si NMR spectra were obtained using a CMX-II spectrometer (Chemagnetics) operating at 8.5 T (71.5 MHz for ^29^Si and 360.1 MHz for ^1^H). A home-built, double-resonance probe with a 5.0 mm (rotor diameter) MAS module was used with spinning speeds up to 7.0 kHz. An initial state of transverse ^29^Si magnetization was created via cross polarization (CP) from spin-locked protons via a variable-amplitude-modification of a Hartmann-Hahn CP match [29,30]. In experiments using a higher MAS speed or performed on samples with a weak heteronuclear dipolar interaction, during the contact interval, the ^1^H spin-lock field was varied linearly, centered on the Hartmann-Hahn match condition (*γ*_H_*B*_1__H_ = *γ*_Si_*B*_1__Si_) and with extremes corresponding to one sideband away (*γ*_H_*B*_1__H_ ± *ω*_MAS_ = *γ*_Si_*B*_1__Si_). The use of CP (either simple or with the variable-amplitude modification) [30,31] has significant effects: (1) The ^29^Si signal from each CP-generated transient is larger than that of a corresponding DP-generated transient; (2) the number of transients acquired per unit time with CP (limited by proton T_1_) is correspondingly increased over that obtained with DP (limited by the typically larger ^29^Si T_1_); and (3) while, for DP ^29^Si NMR each silicon site in the sample typically appears with an intensity (area) proportional to its total population in the sample, provided the recovery time is sufficiently long to permit full spin-lattice relaxation, for ^29^Si signals obtained using CP, only those silicon sites appear spectrally that are in relatively close proximity to hydrogen. Details of the relationship between signal amplitude and internuclear ^29^Si–^1^H distance is determined by details of the pulse timing and any molecular motion present. As the current understanding of the structure of *np*-Si is that hydrogen atoms occur solely on the surface, cross polarization can be considered to be a surface-selective method. High-power ^1^H decoupling (50 kHz) was used in CP ^29^Si experiments, unless otherwise specified. Spectra were obtained by Fourier transformation of complex data after apodization with 71 Hz of Lorentzian line broadening. The π/2 pulse time was 5.0 µs in all cases. For CP experiments the magnetization recovery time and CP contact time (CT) were 2 s and 1 or 14 ms, respectively. ^29^Si chemical shifts are reported in parts per million, referenced to liquid TMS (0.0 ppm), based on substitution of the secondary reference TTMSS (−8.1 and −134.2 ppm relative to TMS).

*^1^H NMR*. Solid-state ^1^H NMR spectra were obtained using a CMX-Infinity spectrometer operating at 14.1 T (600.1 MHz for ^1^H) described above and employing a home-built, single-resonance probe, using a 3.2 mm (rotor diameter) MAS module with a spinning speed of 20 kHz in most cases or using a CMX-II spectrometer operating at 8.5 T (360 MHz for ^1^H), described above (12 kHz MAS speed). An initial state of transverse ^1^H magnetization was created by DP (4.0 µs π/2 pulse). As the probe exhibits a significant ^1^H background (relative to the signal obtained from the samples studied here), it was necessary to experimentally suppress the background when examining the relatively low proton density *np*-Si. This was accomplished by the use of the DEPTH method that utilizes a number of π pulses with various rotating-frame phases to preserve the sample’s legitimate signal (which arises in the more intense rotating-frame RF field in the center of the sample coil) and suppresses the background signal (which arises in the less intense rotating-frame RF field surrounding the sample coil) [32]. An extension of this method used in the work described here permits estimation of the spin-spin relaxation time (T_2_) simultaneously with this background suppression. This method is described in detail in the *Supporting Materials*. Spectra were obtained by Fourier transformation of complex data after apodization with 60 Hz of Lorentzian line broadening. The magnetization recovery time was 3 s. ^1^H chemical shifts are reported in parts per million, referenced to liquid TMS (0.0 ppm), based on substitution of the secondary reference PDMS (0.0 ppm relative to TMS).

*^2^H NMR.* Direct-polarization (DP) solid-state ^2^H NMR spectra were obtained using a CMX-II spectrometer operating at 8.5 T (55.2 MHz for ^2^H). A home-built, single-resonance, transmission-line tuned probe with a 5.0 mm (rotor diameter) MAS module was used with a spinning speed of 7.0 kHz. An initial state of transverse ^2^H magnetization was created by DP (4.0 µs π/2 pulse). No ^1^H decoupling was used. Spectra were obtained by Fourier transformation of complex data after apodization with 20 Hz of Lorentzian line broadening. The magnetization recovery time was 2 s. ^2^H chemical shifts are reported in parts per million, referenced to liquid TMS (0.0 ppm), based on substitution of the secondary reference hexamethylbenzene-*d_6_* (2.2 ppm relative to TMS).

All NMR measurements were made at ambient temperature (~20 °C) without any temperature control.

*NMR Spectrum Simulations.* Each experimental spectrum was analyzed by matching a simulated spectrum made up of a series of contributions, each consisting of a linear combination of frequency-domain and time-domain functions corresponding to Lorentzian and Gaussian functions in the frequency dimension and first-order decaying exponential function in the time dimension. These simulations were carried out using custom-made algorithms, generated in Igor Pro software (Wavemetrics, Inc. Lake Oswego, OR). For each experimental spectrum, the entire experimental and simulated matrices were compared at once, instead of comparing individual slices of the frequency dimension in one step and then comparing the time points obtained from individual comparisons in a second step. Details are given in the *Supporting Materials*.

*Quantum Mechanical Chemical Shift Calculations.* ^29^Si chemical shifts were calculated for various silicon-containing cluster models which potentially can represent models for the surface structure of *np*-Si. The cluster models were created from tetrahedral silicon or silica networks by replacing specific atoms and adjusting the corresponding bond distances. For the initial structures of silicon and silica, the crystalline silicon structure (Si–Si bond length = 0.235 nm) [33] and *β*-cristobalite structure (Si–O bond length = 0.161 nm, Si–O–Si bond angle = 146.4°) were used, respectively. For the H–Si bond length, the silane bond length (0.148 nm) was used [34]. All calculations were carried out with GAUSSIAN software (Gaussian, Inc. Wallington, CT), using the restricted Hartree-Fock (RHF) method with the 6-311 + G(d, p) basis set. Chemical shielding tensors were calculated using the gauge-independent atomic orbital (GIAO) method. Theoretical shielding σ were transformed to relative chemical shifts δ by subtracting the calculated chemical shielding of TMS. Isotropic shieldings were calculated from the relationship σ = (σ_11_ + σ_22_ + σ_33_)/3.

## 3. Results and Discussion

### 3.1. Preliminary np-Si Characterization

The surface area measured for as-received *np*-Si was 86.3 ± 1.1 m^2^ g^−1^. Using the assumption of a spherical particle shape, this value corresponds to an average particle diameter of 40.0 ± 0.5 nm. The surface areas for oxidized and ^1^H_2_O-treated *np*-Si samples were both found to be 86.8 ± 1.1 m^2^ g^−1^.

The total (presumably surface) proton concentration of *np*-Si was estimated by spin counting (carried out by comparing the integral of each ^1^H NMR signal with that of 1,3,5-trimethoxybenzene as an external reference) [35]. For *np*-Si samples evacuated at 150 and 300 °C, this concentration was found to be 0.83 ± 0.07 and 0.82 ± 0.07 mmol H g^−1^, respectively. These values correspond to 5.8 ± 0.5 and 5.7 ± 0.5 H nm^−2^, respectively, based on the surface area measurements reported above.

**Figure 1:**
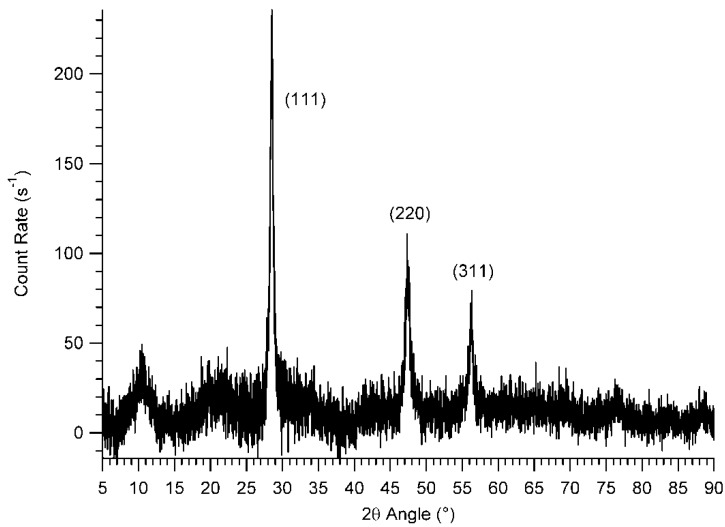
X-ray powder diffraction pattern for as-received *np*-Si, showing crystallographic indices corresponding to each of the crystalline silicon sites.

The X-ray diffraction pattern for as-received *np*-Si is shown in Figure 1, which demonstrates that these particles possess a significant crystalline phase. No significant difference was observed between the angular locations of the signals for *np*-Si and the corresponding values for crystalline silicon. From the width of the (111) signal and using Scherrer’s formula [36], the size of the crystalline phase (which is not necessarily the same as the size of the entire particle) is estimated to be 23 ± 1 nm [36]. This dimension is substantially smaller than both the manufacturer’s reported particle size (50 nm) and the particle size estimated from the BET surface area measurement (40 nm). This discrepancy may be due to some combination of the following: (1) The crystalline silicon region constitutes only a fraction of the entire inhomogeneous particle, a central core, with a surrounding mantle of non-diffracting material (perhaps containing amorphous silicon or amorphous silicon dioxide); (2) there are several diffracting grains or regions within each particle; or (3) the averaging process involved in the diffraction measurement differs substantially from the corresponding average of the surface area measurement.

### 3.2. MAS NMR Studies

**Figure 2:**
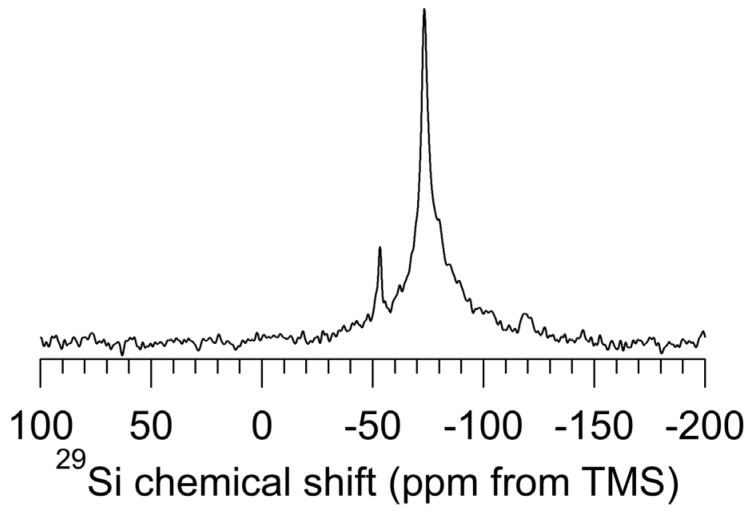
^29^Si (39.7 MHz) DP–MAS (5.6 kHz) NMR spectrum of *np*-Si treated with pentane, then evacuated at 150 °C. An excitation pulse of 30° and a recovery time of 30 s was used and 8000 transients were taken.

Figure 2 shows the ^29^Si DP–MAS NMR (no ^1^H decoupling) spectrum of as-received *np*-Si evacuated at 150 °C. The chemical shift of the dominant signal is centered at −73 ppm, with a line width (full width at half maximum, FWHM) of 4.0 ppm (160 Hz), corresponding closely to the ^29^Si NMR characteristics of *c*-Si [18]. No signal corresponding to *a*-Si (a very broad signal centered at −40 ppm) [18] is seen in this spectrum.

The DP excitation used in obtaining this spectrum does not discriminate *per se* between surface and buried silicon atoms. However, if there is a distribution of ^29^Si T_1_ values and the recovery time used is shorter than three times the largest T_1_ value, then there will be a measurable distortion in the simple relationship of signal intensity to site concentration. The ^29^Si T_1_ of the sharp *c*-Si-like signal (about −73 ppm) in Figure 2 was estimated (by variation the magnetization recovery time) at 14 T to be on the order of 12,000 s (data not shown). This value is comparable to that reported for *c*-Si [18], where it is also reported that ^29^Si T_1_ for *a*-Si ranges from 3000 to 6000 s. Although it would be questionable to specify a lower bound with confidence, if there were a substantial amount of *a*-Si present (e.g., 10 atom % of the silicon) in the *np*-Si sample examined here, one would expect it to be revealed in this spectrum at about −35 ppm [18].

In addition to the sharp peak at −73 ppm in Figure 2, there is also a sharp peak at −52 ppm and a broader peak at −118 ppm (not as reliably detected as the other two peaks). The −73 ppm and −118 ppm peaks are much less intense in Figure 2 than is the −73 ppm peak; however, the relative intensities are highly dependent on the recovery time (between pulses). At recovery times of a few seconds, the intensities of these three peaks are roughly comparable, but at very long recovery times, e.g., thousands of seconds, the −73 ppm peak is heavily dominant. The −52 ppm, −73 ppm and −118 ppm peaks appear to be largely unaffected by removal of ^1^H decoupling; hence, these peaks are due to silicon atoms that are *not* directly bonded to hydrogen atoms. The peak at about −118 ppm is broader than the other two peaks and is not clearly apparent in all the spectra recorded.

The fact that the −52 ppm and −73 ppm peaks are sharp indicates that the corresponding silicon sites are due to geometrically homogeneous sets of structures. In the −73 ppm case, this is readily understood in terms of an assignment to *c*-Si-like silicon regions of the sample.

At recovery times on the order of about 30 s, one sees a broad component at the base of the −73 ppm peak. The breadth of this spectral feature indicates a substantial heterogeneity in chemical structures, probably very similar to the *c*-Si structure, but with small variations of the bond angles and lengths around the *c*-Si values of 109.4 degrees and 0.254 nm, respectively.

In comparing chemical shifts obtained in MAS experiments on solid samples with values reported on the same or similar materials in liquid samples (solutions), one should not be surprised by significant differences. These can result from a combination of sources, including small liquid *vs.* solid structural differences (bond lengths and angles), unaccounted non-nearest-neighbor substituent effects, differences in local and bulk magnetic susceptibility effects, solvent effects and variations in chemical shift referencing methods. For ^29^Si, these differences might be as large as a few ppm.

**Figure 3:**
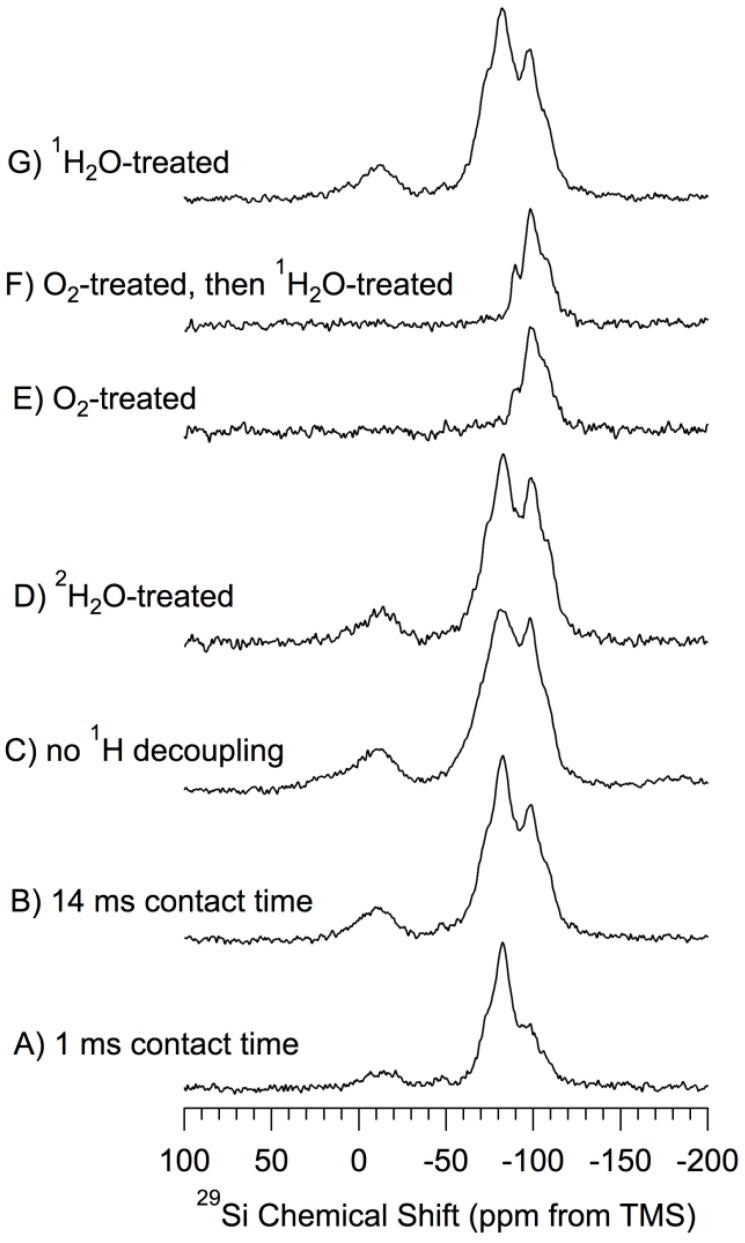
^29^Si (71.5 MHz) CP–MAS spectra of samples based on *np*-Si. ^1^H-decoupled, 7 kHz MAS and 14 ms CP contact time and evacuated at 150 °C, unless noted otherwise. (**A**) 1 ms contact time; (**B**) 14 ms contact time; (**C**) no ^1^H decoupling; (**D**) ^2^H_2_O-treated; (**E**) O_2_-treated (oxidized) at 500 °C; (**F**) O_2_-treated (oxidized) at 500 °C-then-^1^H_2_O-treated; (**G**) ^1^H_2_O-treated.

*^29^Si CP–MAS Results.* Figure 3 shows ^29^Si CP–MAS spectra of a set of samples based on *np*-Si that has been treated in various ways. All of the spectra of Figure 3, except that shown in Figure 3A (1.0 ms CP contact time) were obtained in experiments with a CP contact time of 14 ms. The CP technique is based directly upon dipole-dipole interactions, ^1^H–^29^Si interactions in the present case, and favors ^29^Si NMR signals of silicon atoms that are near hydrogen atoms. The CP–MAS experiments carried out with a long CP contact time (14 ms) may lessen (but do not eliminate) the spatial selectivity of the method.

The ^29^Si spectra of Figure 2 and Figure 3 are so different that, at first glance, they seem to belong to entirely different samples. The spectral differences occur because DP–MAS and CP–MAS are based on completely different mechanisms for generating the ^29^Si spin polarizations that are observed. While the DP–MAS ^29^Si spectrum (Figure 2) relies on ^29^Si spin-lattice relaxation, which in turn is based on time-dependent spin interactions (e.g., the fluctuating fields generated by a small concentration of unpaired electrons), in ^29^Si CP–MAS experiments (Figure 3) the ^29^Si spins derive spin polarization from nearby protons via static components of ^1^H–^29^Si dipolar interactions. Then, in keeping with the popular view that elemental silicon particles are “capped” on the surface by –H and/or –OH moieties, the observed CP *vs*. DP ^29^Si NMR differences can be understood substantially on the basis that the “crystalline core” of a *np*-Si particle consists of crystalline-like silicon sites, most of which are spatially distant from any hydrogen atoms and hence unable to participate effectively in ^1^H– > ^29^Si CP. This “crystalline core” is surrounded by a hydrogen-containing, amorphous sheath in which the ^29^Si nuclei can participate more or less efficiently in ^1^H– > ^29^Si CP. One can envision two extreme cases of a material with a chemically homogeneous crystalline portion and a chemically inhomogeneous amorphous portion: (a) A series of distinct particles, each with a crystalline core surrounded by an amorphous (surface) mantle; or (b) a cluster of crystalline cores surrounded by an amorphous portion that connects the cores via some type of covalently bound amorphous network (the surface region)—like whole peanuts in chunky peanut butter.

Taking the typical *np*-Si particle diameter of about 50 nm, if one makes the oversimplified assumption of a spherical particle shape, a simple calculation yields the result that about 6% of the idealized *np*-Si particle is “at the surface”, *i.e.*, within about 0.5 nm of the outer edge of the particle. According to this interpretation, the DP–MAS ^29^Si spectrum of Figure 2 is dominated by the silicon sites of the crystalline core of the particle, with the CP–MAS ^29^Si spectra of Figure 3 representing the roughly 6% of the silicon sites that are “at the surface”.

There must, of course, be an *interface region*, presumably not more than a couple of silicon atoms in “thickness”, between the core (crystalline) region and the surface region. Our view of the interface region is one in which the silicon atoms are all covalently bonded to four other silicon atoms, therefore having local electronic distributions (and, hence, ^29^Si chemical shifts) that are very similar to the corresponding properties of crystalline silicon (*i.e*., close to −73 ppm), but close enough in physical proximity to the nearest hydrogen atoms to be able to participate marginally in ^1^H– > ^29^Si CP (requiring a long CP contact period). Presumably, the silicon atoms of the interface region will have local chemical structures of the types, (*Si*–)_3_**Si**–*Si*–H, (*Si*–)_3_**Si**–*Si*–OH, (*Si*–)_3_**Si**–*Si*–O–*Si*–H and (*Si*–)_3_**Si**–*Si*–O–*Si*–OH, where again it is to be understood that all silicon atoms represented by *Si* are actually four-coordinate.

For purposes of chemically-focused spectral interpretation, each of the spectra of Figure 3 was simulated in terms of a set of peaks with line shapes that were either Gaussian (−14 ppm contribution) or Lorentzian (all other contibutions). For each of the spectra of Figure 3, the use of a mixed (linear combination of Lorentzian and Gaussian) line shape for any of the contributions did not improve the match between the experimental and simulated spectra. Pictorial details of these simulations are given in the *Supporting Materials.* Although the quality of the match between an experimental and a simulated spectrum, as represented by the weak intensity of the difference spectrum, is very good, one should, nevertheless, keep in mind the fact that, as is the case in such simulations of most functions with severely overlapping contributions (“peaks”), there is a substantial degree of arbitrariness in this kind of procedure, especially when signal-to-noise is limited, as it is in the present case. The relative intensities of the spectral contributions resulting from the simulations pictured in the *Supporting Materials* are summarized in Table 1 (line widths in *Supporting Materials*). One should note that quantitatively significant intensity information, even in a relative sense, is not necessarily achieved from CP spectra obtained at just one CP contact time. For true quantitation a variable contact-time study is usually required. Hence the relative signal intensities reported in Table 1 should be considered to be only *qualitative* indications of relative populations.

One should also note that no ^29^Si NMR intensity in the crystalline silicon region around −73 ppm is included in the results summarized in Table 1. However, as indicated above, there must be at least a small core-like contribution of the interface region in the CP–MAS results, especially those obtained at a CP contact time of 14 ms, a contribution that presumably is roughly comparable in intensity to that of the surface region. Accordingly, one can readily include a 4.0 ppm wide spectral contribution at about −73 ppm in the simulations, and still achieve an excellent fit with each experimental spectrum. However, it *is not necessary* to include such a contribution to achieve a high-quality fit. The inclusion of this simulation contribution is accompanied by a substantial reduction in the contribution centered at about −83 ppm, typically amounting to 25%–50% of the −83 ppm intensity contribution.

**Table 1:** Parameters Used in the Simulations of ^29^Si CP–MAS Spectra of Figure 3 ^a^.

			Signal Intensity ^b^				
Sample ^c^/^29^Si Chemical Shift (ppm) ^d^	−14	−74	−83	−89	−99	−109	Total ^e^
1 ms CT (Figure 3A)	73	74	250	57	92	24	540
14 ms CT (Figure 3B)	170	170	270	85	240	65	1000
^1^H coupled (Figure 3C)	150	160	390 ^f^	45	220	43	1004
^2^H_2_O treated (Figure 3D)	150	155	375	30	320	100	1130
500 °C O_2_ (Figure 3E)	0	0	0	20	180	80	280
500 °C O_2_, then ^1^H_2_O treated (Figure 3F)	0	0	0	30	200	80	310
^1^H_2_O treated (Figure 3G)	160	200	330	58	260	72	1080

^a^ Unless otherwise specified, all spectra were obtained with a CP contact time (CT) of 14 ms. Some details about the deconvolutions are given in the *Supporting Materials*; ^b^ Integrated signal intensity of this deconvolved spectral contribution. Uncertainties estimated to be, for each case, about +10% of the corresponding reported intensity; ^c^ Sample preparation or important parameter of the NMR experiment. Samples of A, B and C were *np*-Si that had been pentane-treated and evacuated at 150 °C. Spectra E–G were all obtained with CT = 14 ms; ^d^ Values in ppm (relative to TMS) refer to the position of the center of each spectral contribution represented; ^e^ Total (spectrum-wide) integrated intensity for this spectrum, normalized to 1000 for the spectrum of Figure 3B; ^f^ 24% of the signal intensity of this peak is in MAS sidebands.

**Figure 4:**
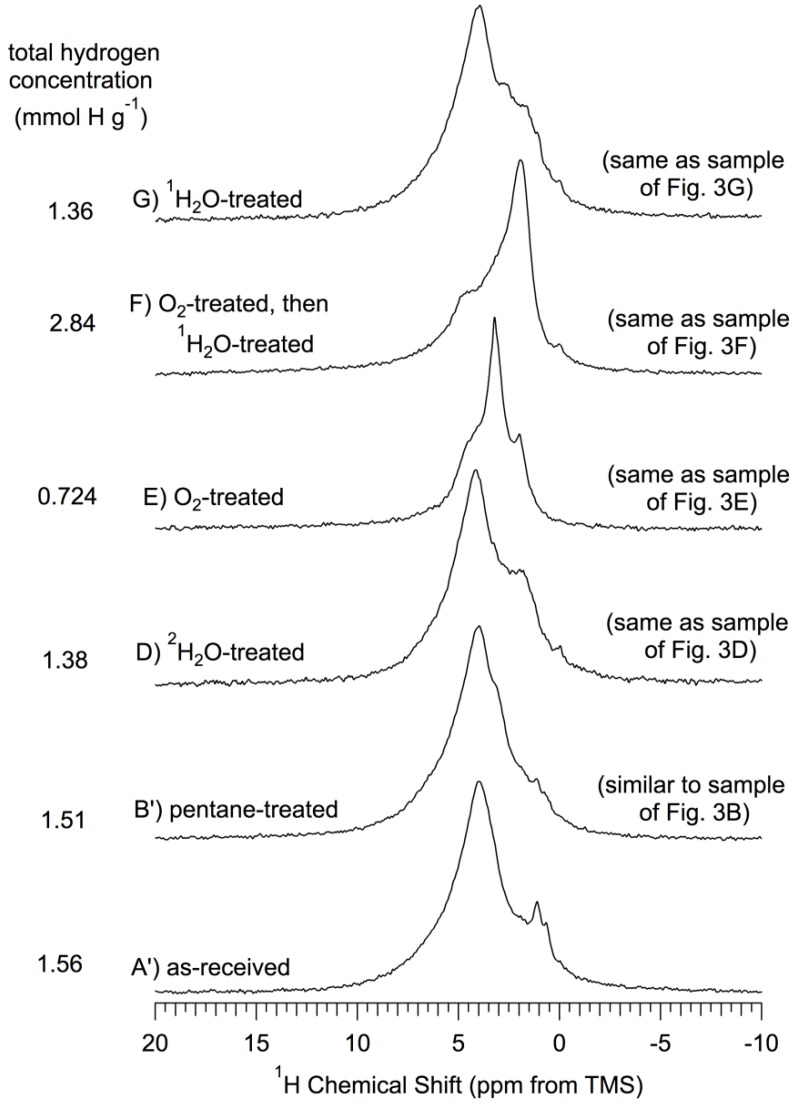
^1^H (360 MHz) MAS (15–17 kHz) NMR spectra of *np*-Si and treated *np*-Si samples. All samples were evacuated at 150 °C *in vacuo* after the treatment described and prior to NMR spectrum collection, as described in the text. (**A’**) As-received *np*-Si; (**B’**) Pentane-treated-then- evacuated at 150 °C (similar to the sample of Figure 3B, which was *not* pentane treated); (**D**) ^2^H_2_O-treated-then-evacuated at 150 °C (same as sample of Figure 3D); (**E**) O_2_-treated (oxidized) at 500 °C, -then-evacuated at 500 °C (same as sample of Figure 3E); (**F**) O_2_-treated (oxidized) at 500 °C-then-^1^H_2_O-treated-then-evacuated at 150 °C (same as sample of Figure 3F); (**G**) ^1^H_2_O-treated-then- evacuated at 150 °C (same as sample of Figure 3G). All spectra scaled so that the total area is proportional to the total hydrogen concentration for each sample.

**Figure 5:**
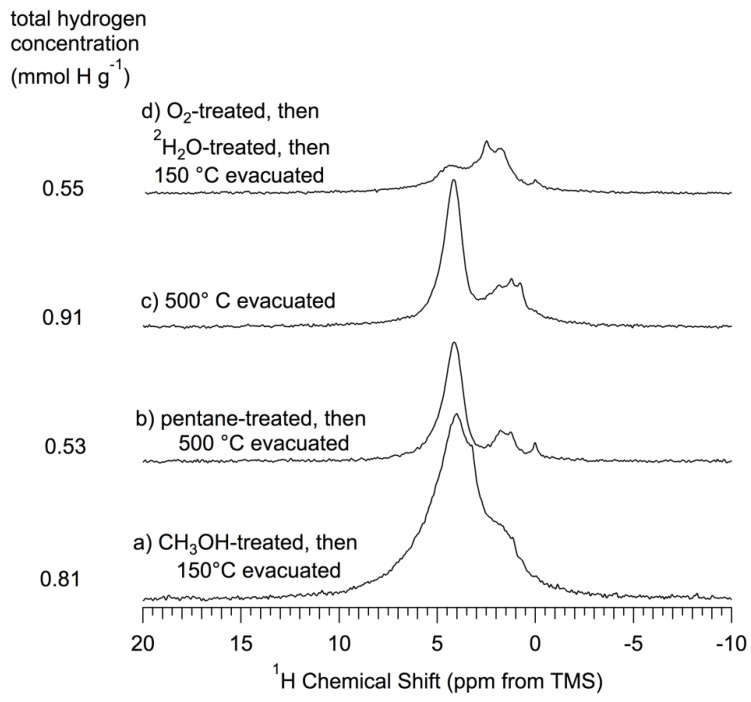
^1^H (360 MHz) MAS (15–17 kHz) NMR spectra of *np*-Si and treated *np*-Si samples. (**a**) CH_3_OH-treated; (**b**) Pentane-treated-then-evacuated at 500 °C; (**c**) Evacuated at 500 °C; (**d**) O_2_-treated (oxidized) at 500 °C-then-^2^H_2_O-treated-then-evacuated at 150 °C. All spectra scaled so that the total area is proportional to the total hydrogen concentration for each sample.

*^1^H MAS* *Results*. 360 MHz ^1^H MAS experiments were carried out, using 15–17 kHz spinning, on a variety of samples based on *np*-Si. The spectra are collected in Figure 4 and Figure 5. For the reasons given above for caution in comparing MAS-determined chemical shifts with literature values (especially for liquids), one might expect analogous differences here, perhaps up to about 1.0 ppm. ^1^H MAS spectra of samples that correspond to the ^29^Si spectra of Figure 3 are shown in Figure 4; these figures also show the total ^1^H content per gram for each sample, as described above.

Examination of the ^1^H MAS spectra in Figure 4 reveals that there is ^1^H NMR intensity—as peaks, shoulders or “wings”—at chemical shifts spanning the range from about 7 ppm to about 0 ppm. These spectra and those of Figure 5 were analyzed via deconvolution/simulation based on up to nine peaks or contributions; not all nine components are represented in any one spectrum. Table 2 collects the results of these simulations, the details of which can be found in the *Supporting Materials*.

**Table 2:** Summary of the signal areas of the simulation/deconvolutions of ^1^H MAS spectra of Figure 4 and Figure 5.

	Signal Integrated Intensity ^a^									
Sample ^b^/^1^H Chemical Shift (ppm) ^c^	6.0	4.8	4.5	3.9	3.3	3.1	2.2	1.1	0.0	Total ^d^
CH_3_OH (Figure 5a)	44	31	48	32	35	7.0	21	26	0.0	244
oxidized, then ^2^H_2_O (Figure 5d)	16	18	12	10	17	0.0	37	54	5.0	164
oxidized, then ^1^H_2_O (Figure 4F)	11	39	21	16	17	0.0	45	62	2.0	213
^2^H_2_O (Figure 4D)	75	75	70	40	39	0.0	39	75	4.0	417
^1^H_2_O (Figure 4G)	60	48	84	48	48	0.0	48	72	3.0	411
pentane, then 500 °C evacuated (Figure 5b)	13	19	86	11	0.0	0.0	0	26	7.0	162
500 °C evacuated (Figure 5c)	13	17	122	0.0	0.0	0.0	9	103	13	277
500 °C oxidized (Figure 4E)	23	19	15	23	89	0.0	15	31	2.0	217
pentane, then 150 °C evacuated (Figure 4B’)	93	46	110	76	56	0.0	32	41	0.0	454
as-received (Figure 4A’)	60	42	68	101	130	0.0	40	26	3.0	470

^a^ Integrated signal intensity of this deconvolved spectral contribution. Uncertainties estimated to be, for each case, about ±10% of the corresponding reported intensity; ^b^ Sample preparation or important parameter of the NMR experiment. Sample identities defined in Figure 4 and Figure 5; ^c^ Values in ppm (relative to TMS) refer to the position of the center of each spectral contribution represented; ^d^ Total (spectrum-wide) integrated intensity for this spectrum, normalized to 1000 for the spectrum of Figure 3B.

*Assignments and Quantum Mechanical Calculations of ^29^Si Chemical Shifts.* As an aid in identifying specific ^29^Si chemical shifts with specific structural moieties, ^29^Si chemical shift calculations were performed, using the GAUSSIAN program, on model clusters that represent possible local structures that may contribute to the overall surface structure of *np*-Si. The results, which cover an experimental range of about 70 ppm, are summarized in Table 3. As a prior calibration of the ^29^Si chemical shift calculations on model surface clusters, ^29^Si chemical shifts were calculated on several Si-containing molecules (molecules 1–8 in Table 3) and compared with the values obtained experimentally. In the interest of saving computational time in the calculations on possible structural models for specific surface sites of *np*-Si, the SiOSiH_3_ moiety was used instead of SiOSi(–O–*Si*)_3_ to represent a **Si**–O–*Si* bridge between the silicon atom for which δ^29^Si is calculated (represented in bold face) and another silicon atom in the structure. The relatively close agreement for model compounds 6, 7 and 8 in Table 3 with experimental results on silica gel indicates that this is a reasonable approximation for this kind of structure.

**Table 3:** Quantum chemically calculated and experimentally obtained ^29^Si chemical shifts ^a^.

	Molecule	□ ^29^Si (ppm) Experimental Calculated			Molecule	^29^Si(ppm) Calculated
1	SiH_4_	−93 ^b^	−91	11	HSi(–SiH_3_)_2_–OSiH_3_	−18
2	H_3_Si–SiH_3_	−103 ^c^	−96	12	(HO–)Si(–SiH_3_)_3_	−14
3	H_2_Si(–SiH_3_)_2_	−115 ^c^	−105	13	(HO–)_2_Si(–SiH_3_)_2_	−12
4	HSi(–SiH_3_)_3_	−136 ^c^	−124	14	H_2_Si(–OSiH_3_)_2_	−44
5	Si(–SiH_3_)_4_	−165 ^c^	−159	15	H_2_Si(–SiH_3_)–OSiH_3_	−37
6	(HO–)_2_Si(–OSiH_3_)_2_	−89 ^d^	−86	16	(HO–)Si(–SiH_3_)_2_–OSiH_3_	−2
7	HO–Si(–OSiH_3_)_3_	−99 ^d^	−93	17	(HO–)Si(–SiH_3_)(–OSiH_3_)_2_	−49
8	Si(–OSiH_3_)_4_	−109 ^d^	−100	18	(HO–)_2_Si(–SiH_3_)–OSiH_3_	−37
9	HSi(–OSiH_3_)_3_	–	−77	19	HO(H)Si(–SiH_3_)_2_	−22
10	HSi(–SiH_3_)(–OSiH_3_)_2_	–	−14	20	HO(H)Si(–SiH_3_)–OSiH_3_	−24
				21	HO(H)Si(–OSiH_3_)_2_	−71

^a^ ^29^Si Chemical shifts of Si atom in bold face; ^b^ Reference [37]; ^c^ Reference [38]; ^d^ Reference [39] and reference [40].

For the cases in which calculated results can be compared with reliable experimental data, it seems that the calculated ^29^Si chemical shifts are commonly more positive (lower shielding) by a few ppm than those obtained experimentally. From the results for compounds 1–9 in the table, a plot of δ^29^Si(calculated) *vs.* δ^29^Si(experimental) shows a very smooth correlation (not shown here).

Silicon-containing compounds that might represent the structures of sites on a *np*-Si surface are included in Table 3. The model cluster H–**Si**(–OSiH_3_)_3_ (molecule 9) represents hydrogen-terminated silicon atoms bonded to three bridging oxygen atoms, H–**Si**(–O*Si*)_3_; and the ^29^Si chemical shift for the model cluster is calculated to be −77 ppm. This chemical shift is close to the experimentally observed chemical shift of the contribution/signal at about -80 ppm, which is assigned below to some kind(s) of Si(–H)*_n_* moiety, specifically −83 ppm for (*Si*–O–)_3_**Si**–H and −74 ppm for (*Si*–*O*–)_2_**Si**(H)OH. The experimentally observed −74 ppm chemical shift is close to the result calculated for molecule 21 in the table (−71 ppm), providing support for this chemical shift assignment to the HO(H)**Si**(–O–*Si*)_2_ structure (*vide infra*).

Among the calculated results of Table 3, only those for model molecules 10, 11, 12, 13, 16, 19 and 20 are within 20 ppm of −14 ppm, the position of the broad, low-intensity signal in Figure 3. Of these seven model molecules in Table 3, numbers 10, 11, 19 and 20 are tentatively ruled out because they contain **Si**–H bonds, which is shown below to be an unlikely assignment. This leaves, as possible assignments, the structures, HO–**Si**(–*Si*)_3_, (HO–)_2_**Si**(–*Si*)_2_ and HO–**Si**(–*Si*)_2_(–O–*Si*), which are modeled in Table 3 by model molecules 12, 13 and 16, respectively; these three structural possibilities are all covered by the formula, (HO)*_n_***Si**(–*Si*)*_m_*(–O*Si*)_4−*n*−*m*_, which is taken as the structural assignment for the −14 ppm signal. The breadth of the −14 ppm peak may indicate that more than one of these structures contributes to this signal.

Table 4 summarizes the ^29^Si chemical shift assignments that are employed in this paper. Some of these assignments are taken from a critical examination of the literature and are supported by this study.

**Table 4:** ^29^Si chemical shift assignments of this study ^a^.

δ ^29^Si (ppm)	Molecule	δ ^29^Si (ppm)	Molecule
−14	(HO)_n_Si(–Si)_m_(–OSi)_4−n−m_	−89	(Si–O–)_2_Si(–OH)_2_^e,f^
−74	(Si–O–)_2_Si(H)OH ^b,c^	−99	(Si–O–)_3_SiOH ^e,f^
−73	Si(–Si)_4 cryst__al_^d^ + interface	−109	(Si–O–)_4_Si ^e,f^
−83	(Si–O–)_3_Si–H ^b,c^	–	–

^a^ Taken from a critical examination of the literature and the experiments and calculations of this study; ^b^ Reference [41]; ^c^ Reference [42]; ^d^ Reference [18]; ^e^ Reference [43]; ^f^ Reference [44].

### 3.3. Chemical Interpretations

*“Untreated” np-Si samples.* Figure 3B shows the proton-decoupled ^29^Si CP–MAS NMR spectrum of *np*-Si evacuated at 150 °C. The simulated spectrum of Figure 3B (*Supporting Materials*) consists of six heavily overlapping signals, each with a unique chemical shift, whose spectral characteristics are summarized in Table 1. The signal at −14 ppm, in addition to differing from the other contributions in line shape, also differs from the rest in the spectrum simulation of Figure 3B in having a significantly larger line width of 28 ppm, compared with 9 to 12 ppm for the others. This suggests that the −14 ppm signal (signal/contribution) likely represents more than one site structure on the surface. This −14 ppm signal appears in all of the spectra of Figure 3 for samples that have *not* been subjected to the oxidative treatment with O_2_ at 500 °C. No spinning sidebands are observed for any signal. It is worth noting that −74 ppm and −83 ppm are very close to the −73 ppm and −83 ppm values that have been reported for (*Si*–O–)_2_**Si**(H)OH and (*Si*–O–)_3_**Si**–H structures, respectively, from data on solid and/or liquid samples of (R–O–)_3_Si–H or its polysiloxane polymers and/or modified silicas [41,42]. Here the symbol, *Si*, stands for silicon atoms that are bonded or bridged to the silicon atom, **Si**, on whose chemical shift we are focused, and it is understood that *Si* has additional bonds that are not shown in the formula.

In examining the ^29^Si CP–MAS results and attempting to assign specific spectral regions to specific local surface structures, an important strategy is to establish which spectral regions are identified with silicon atoms that have directly attached (bonded) hydrogen atoms, *i.e.*, with Si–H bonds. In solid-state ^29^Si NMR, one is drawn to experimental approaches that depend predictably on the strengths of ^1^H–^29^Si dipole-dipole interactions. One such approach is to examine the effect of changing the CP contact time (CT), since the rate of ^1^H– > ^29^Si CP transfer of polarization depends strongly on the strength of pertinent ^1^H–^29^Si dipolar interactions, which in turn are inversely proportional to the cube of the ^1^H–^29^Si internuclear distance.

Comparison of the spectra of Figure 3A,B, which come from spectra obtained with 1 ms and 14 ms, respectively, provides an opportunity to assess *qualitatively* the CP dynamics. Of course, to characterize the CP dynamics *quantitatively*, one would need a more extensive study of the relevant spin dynamics (most commonly, a variable-contact-time study). To the extent that the 14 ms spectra represent, at least qualitatively, the relative site populations of the surface region (and interface region), the integrated intensities shown in Table 1 indicate that the surface populations, identified in terms of ^29^Si chemical shift, fall in the order: −83 ppm > −99 ppm > −14 ppm ≈ −74 ppm > −89 ppm > −109 ppm. As expected, in the comparison highlighted here, seen both in the spectra and in the corresponding parameters in Table 1 that were derived from them, the overall integrated spectral intensity of the spectrum measured with CT = 14 ms (Figure 3B) is substantially larger than for the spectrum obtained with CT = 1 ms (Figure 3A). This intensity difference is very strong for the spectral contributions at around −14 ppm, −89 ppm, −99 ppm and −109 ppm, and not so large for the contribution at −83 ppm. This pattern seems reasonable if one assumes that the −83 ppm contribution comes from Si–H sites, for which CP transfer will be well developed already at 1 ms of CP contact, and will have less to gain from a CT increase to 14 ms than will CP intensity at silicon atoms with no directly bonded hydrogen. The CP responsible for those spectral contributions, at −14 ppm, −89 ppm, −99 ppm and −109 ppm, benefit more markedly from the longer CP CT; therefore, the corresponding silicon sites center on silicon atoms with no directly-bonded hydrogens. The −74 ppm spectral contribution, for reasons not yet understood, also shows a strong response to increasing CT. The ratio of integrated spectral intensities obtained with CT values of 1 ms and 14 ms, also given in Table 1, are consistent for the spectral contributions at −89 ppm, −99 ppm and −109 ppm with results published on fumed silica [44] and silica gel [45], derived from variable-contact-time data for silica peaks identified as (*Si*–O–)_2_**Si**(OH)_2_, (*Si*–O–)_3_**Si**OH and (*Si*–O–)_4_**Si** contributions, respectively.

Figure 3C shows the ^29^Si CP–MAS NMR (not ^1^H decoupled) spectrum of pentane-treated *np*-Si evacuated at 150 °C. The spectrum was recorded *without proton decoupling*. Even without proton decoupling, the ^29^Si–^1^H dipolar coupling will be largely averaged by 7 kHz MAS, but the ^29^Si–^1^H J coupling will still be operative, as confirmed by simulations carried out with the Simpson program [46]. Comparing the spectra of Figure 3B,C, and the corresponding parameters in Table 1, one sees that the line widths of the spectral contributions at about −74 ppm and −83 ppm have roughly doubled when the proton decoupler was turned off, while the line widths of the −14 ppm, −89 ppm, −99 ppm and −109 ppm spectral contributions are almost unchanged. The large increases in line widths observed are likely due to a combination of residual dipolar broadening and unresolved ^29^Si–^1^H J coupling in silicon sites with directly-bonded hydrogens. J_Si–H_ in silatrane, N(CH_2_CH_2_O)_3_Si–H, is reported to be 270 Hz [47]. These patterns are consistent with the following tentative assignments: −14 ppm, no directly-bonded hydrogens (at least for the main structural constituent contributing to this spectral region); −74 ppm, (*Si*–O–)_2_**Si**(H)OH (substantial uncertainty associated with the CT dependence described above); −73 ppm, interface sites; −83 ppm, (*Si*–O–)_3_**Si**–H; −89 ppm, (*Si*–O–)_2_**Si**(OH)_2_; −99 ppm, (*Si*–O–)_3_**Si**–OH; −109 ppm, (*Si*–O–)_4_**Si**.

**Figure 6:**
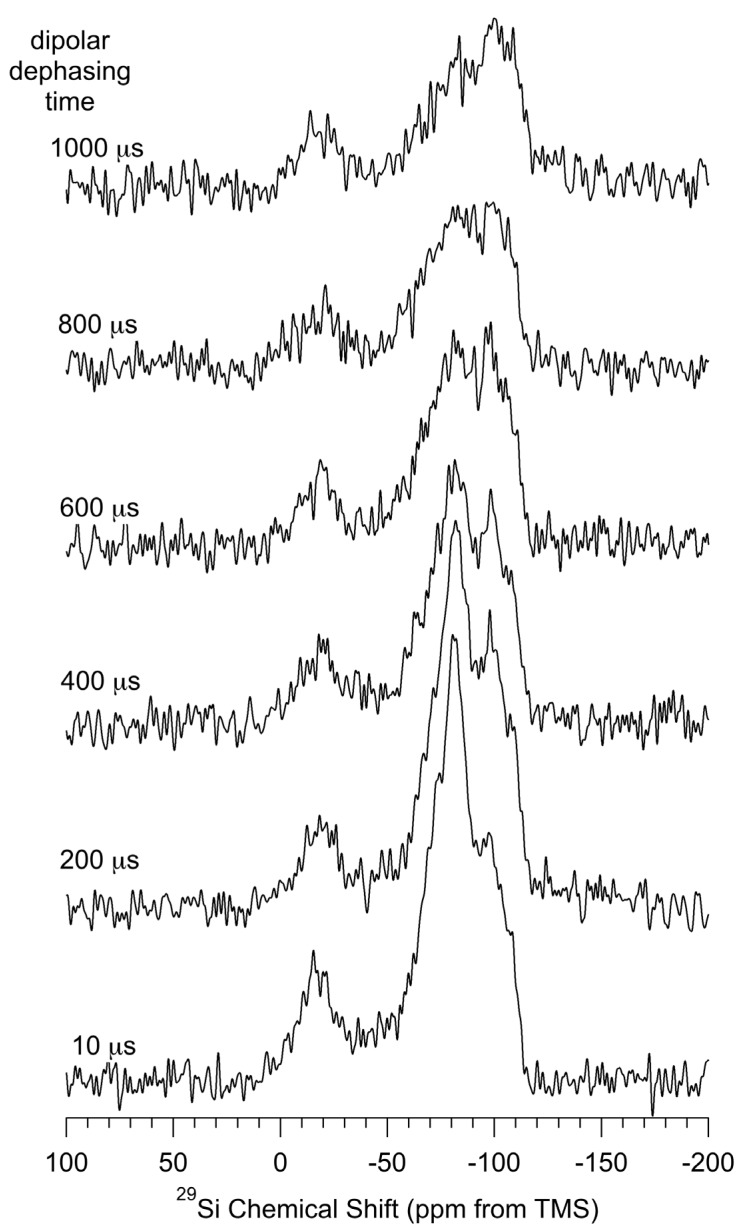
Dipolar-dephasing ^29^Si (71.5 MHz) CP–MAS (5.0 kHz) spectra of *np*-Si for various dipolar-dephasing periods, as indicated.

Another dipole-based technique that is used routinely in ^13^C MAS NMR to establish C, H proximity (assuming rapid MAS or atomic-level motions do not largely average the relevant dipole-dipole interactions), is the interrupted decoupling or *dipolar dephasing* technique [48]. In this technique a period, often about 40 µs, is inserted between the end of the period generating ^13^C magnetization from CP and the beginning of proton-decoupled ^13^C detection. During this “interrupt” period, ^13^C magnetization of carbons that are strongly impacted by dipolar interaction(s) with one or more protons (e.g., directly-bound ^13^C–^1^H in relatively rigid structures) is rapidly dephased and strongly attenuated in the observed ^13^C spectrum. Figure 6 shows the result of applying the dipolar-dephasing technique to the CP–MAS ^29^Si spectrum of *np*-Si evacuated at 150 °C. From the spectra shown for various durations of the dephasing period, one sees that magnetization of the region around −80 ppm (including both the −74 ppm and −83 ppm spectral contributions) dephases much more rapidly than for other regions of the spectrum, indicating that the ^29^Si resonance(s) giving rise to spectral intensity in that region are due to silicon sites that are in closest proximity to H, e.g., Si(–H)*_n_* sites.

The integrated intensities of each contribution in the deconvolutions of the spectra in Figure 6 (details in the *Supporting Materials*) for each dephasing period (interrupt period) were analyzed in terms of a single decaying exponential function (almost certainly an oversimplification); this analysis yielded the following time constants describing the decays of the various ^29^Si CP–MAS spectral contributions: −14 ppm, 1.8 ms; −74 ppm, 1.0 ms; −83 ppm, 0.60 ms; −89 ppm, 1.0 ms; −99 ppm, 3.5 ms; −109 ppm, 5.0 ms. The dipolar-dephasing time constants for the −89 ppm and −99 ppm contributions are in reasonable agreement with published values for the (*Si*–O–)_2_**Si**(–OH)_2_ and (*Si*–O–)_3_**Si**–OH signals, respectively, derived from dipolar-dephasing experiments on fumed silica [44] and are in the same order (albeit with much smaller values) as are dipolar-dephasing constants derived from silica gel data [45].

The six dipolar-dephasing time constants given above are qualitatively consistent with what one would expect in terms of the chemical shift assignments suggested above and in interpretations below, and summarized in Table 4, *i.e.*, (HO–)*_n_***Si**(–*Si*)*_m_*(–O–*Si*)_4−*m−n*_ for −14 ppm, (*Si*–O–)_2_**Si**(H)OH for −74 ppm, interface sites for −73 ppm, (*Si*–O–)_3_**Si**–H for −83 ppm, (*Si*–O–)_2_**Si**(–OH)_2_ for −89 ppm, (*Si*–O–)_3_**Si**–OH for −99 ppm and (*Si*–O–)_4_**Si** for −109 ppm. The last three structures correspond to silica-like surface sites.

The ^1^H MAS spectra of as-received *np*-Si (Figure 4A’) and *np*-Si that has been treated with pentane-then-evacuated at 150 °C (Figure 4B’) consist of a broad peak centered at about 4 ppm, with some intensity in the regions around 3.3 ppm, 2.2 ppm, 1.1 ppm and 0.7 ppm; the overall (spectrum-wide) intensities for the two samples are essentially the same. Such patterns (aside from the small features at 0.7 ppm, which are not included in the simulations from which Table 2 was generated) are represented by substantial numbers for several contributions in the deconvolution results given in Table 2. The samples evacuated at 500 °C (Figure 5b,c) show a sharpening of the main pattern that includes a maximum at about 4.3 ppm and a more sharply defined intensity pattern in the 0 to 2 ppm range.

^1^H MAS spectra of the *np*-Si samples that had been evacuated at 500 °C show substantial decreases in overall spectral intensity, more than 1/3 for the 500 °C-evacuated sample (Figure 5c) and about 2/3 for the pentane-treated-then-500 °C-evacuated sample (Figure 5b) relative to spectra of the samples that have not been heated at 500 °C (Figure 4A’,B’). One could speculate that this ^1^H NMR intensity loss centered around 4 ppm could be due to some kind of dehydrogenation reaction of the type represented in Equation 1 (where it is understood that the symbol, *Si*, stands for a silicon atom to which there are additional bonds that are not shown explicitly) or a condensation process of the types represented in Equation 2 or more likely, Equation 3,

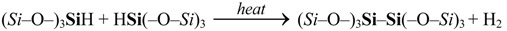
(1)

(*Si*–O–)*_n_*(*Si*–)_3−*n*_**Si**–H + HO–**Si**(–*Si*)_3−*p*_(–O–*Si*)*_p_* → (*Si*–O–)*_n_*(*Si*–)_3*−n*_**Si**–**Si**(–*Si*)_3−*p*_(–O–*Si*)*_p_* + H_2_O (2)

(*Si*–O–)*_n_*(*Si*–)_3−*n*_**Si**–OH + HO–**Si**(–*Si*)_3−*p*_(–O–*Si*)*_p_* → (*Si*–O–)*_n_*(*Si*–)_3−*n*_**Si**–O–**Si**(–*Si*)_3−*p*_(–O–*Si*)*_p_* + H_2_O (3)

Processes of these types could also account for the loss of CP–MAS ^29^Si intensity centered at about −80 ppm, identified above as sites of the type, (Si–O–)_3_**Si**–H (or (*Si*–O–)_2_**Si**(H)OH), in the spectra of *np*-Si that has been heated at 500 °C in the presence of O_2_ (*vide infra*); however, this change in spectra associated with O_2_ treatment at 500 °C could also be associated with an oxidative process of the types represented in Equations 4 and 5.

(*Si*–O–)_3_**Si**–H + HO–**Si**(H)( –O–*Si*)_2_ + 1/2O_2_ → (*Si*–O–)_3_**Si**–O–**Si**(H)(–O*Si*)_2_ + H_2_O (4)

(*Si*–O–)_3_**Si**–H + H–**Si**(OH)(–O–*Si*)_2_ + 1/2O_2_ → (*Si*–O–)_3_**Si**–**Si**(OH)(–O*Si*)_2_ + H_2_O (5)

**Figure 7:**
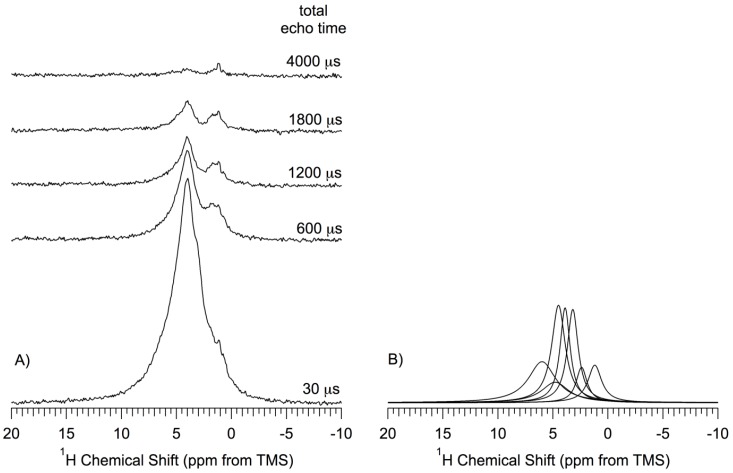
(**A**) ^1^H (360 MHz) MAS (14.3kHz) Depth-Echo spectra of a *np*-Si sample evacuated at 150 °C as a function of total echo time, *τ*_total_ = 2*τ*_1_ + 2*τ*_2_. Spectra were obtained by the DEPTH-echo pulse sequence with 1024 transients, shown without amplitude scaling (true amplitude); (**B**) Deconvolution/simulation contributions to the ^1^H NMR spectrum obtained at *τ*_total_ =30 μs.

Figure 7 shows the ^1^H MAS NMR spectra, obtained by the DEPTH-echo sequence, of the *np*-Si sample (pentane-treated-then-evacuated at 150 °C) as a function of total echo time (*τ*_total_ = 2*τ*_1_ + 2*τ*_2_). In this technique, details of which are given in the *Supporting Materials*, probe background signals are suppressed and the isotropic part of the ^1^H NMR chemical shift refocuses for any time *τ*_total_; for low MAS speed, the anisotropic part of the ^1^H chemical shift can refocus completely only when *τ*_total_ is equal to 2n*τ*_r_, where n is an integer and *τ*_r_ is the MAS rotor period, but is effectively averaged for all *τ*_total_ if the MAS speed is much larger than the CSA (as in the present case, with MAS speeds of at least 12 kHz). The inhomogeneous part of dipolar interactions (^1^H–^1^H and ^1^H–^29^Si) are also refocused, along with the chemical shift effects, each time that every refocusing interval equals an even number of rotor periods (2n*τ*_r_). Thus, it is the *homogeneous* contribution of dipolar interactions in effect over the *τ*_total_ period that preferentially attenuates the transverse magnetization of the protons that are most strongly involved in homogeneous dipolar interactions. This is roughly analogous to the well-known dipolar dephasing technique applied routinely in solid-state ^13^C NMR and represented in Figure 6 for ^29^Si NMR, or to CRAMPS ^1^H experiments with dipolar dephasing [49,50]. In obtaining the spectra shown in Figure 7, the parameter *τ*_total_ was varied from 30 µs to 4000 µs, keeping *τ*_total_ equal to 2n*τ*_r_. Only spectra obtained for *τ*_total_ = 30, 600, 1200, 1800 and 4000 µs are shown in Figure 7.

The ^1^H NMR spectra obtained with various *τ* values were deconvolved into seven Lorentzian-shape peaks, centered at 6.0 ppm, 4.8 ppm, 4.5 ppm, 3.9 ppm, 3.3 ppm, 2.2 ppm and 1.1 ppm (shown in Figure 7B only for *τ*_total_ = 30 µs). According to this deconvolution process, the corresponding magnetization decay of each of the seven contributions was represented by an exponential function with a time constant, *T*_2_’ estimated by fitting each measured intensity to a separate exponential decay (details in the *Supporting Materials*). The *T*_2_’ values obtained from this process are: 6.0 ppm, 0.50 ms; 4.8 ppm, 0.50 ms; 4.5 ppm, 0.50 ms; 3.9 ppm, 0.90 ms; 3.3 ppm, 0.60 ms; 2.2 ppm, 0.40 ms; 1.1 ppm, 2.0 ms. The relative area (integrated intensity) of each of the seven peaks is reflective of the (NMR-visible) abundance of the species (subject to the spectroscopic conditions identified earlier) giving rise to the peak. The observed relative abundances are: 4.5 ppm > 6.0 ppm > 3.9 ppm > 3.3 ppm > 4.8 ppm > 1.2 ppm > 2.2 ppm. The relative individual areas range from 24% to 7% of the total area.

As can be seen in Figure 7A and the T_2_’ values given above, the lowest-shielding ^1^H MAS contribution decays by dipolar dephasing faster than do the higher-shielding contributions, which indicates that the protons that contribute to the lower-shielding side of the spectrum are involved in stronger homogeneous ^1^H–^1^H dipolar effects than those on the higher-shielding side. If the 1.2 ppm peak is assigned to protons of isolated hydroxyl groups, then its larger *T*_2_’ value can perhaps be explained partially by the motion of those protons about a Si–O bond. It has been shown previously that hydroxyl groups of a silica gel surface execute fast (with reference to a 5 × 10^−6^ s time scale defined by deuterium quadrupole interactions) [51] limited-extent rotational (librational) diffusion about the Si–O axis; the librational motions can be restricted by hydrogen bonds. The librational motions of *isolated* hydroxyl groups are less restricted than those of hydrogen-bonded hydroxyls (the ^1^H–^1^H distance between a pair of isolated hydroxyl groups is longer than that of a hydrogen-bonded hydroxyl pair), which results in smaller ^1^H–^1^H dipolar interactions for the isolated hydroxyls.

The small 2.2 ppm contribution in Figure 7B can tentatively be assigned to protons of hydrogen-bonded hydroxyls. It is known that hydrogen bonding produces a proton chemical shift to lower shielding and that the magnitude of this effect increases with stronger hydrogen bonding [51,52]. Thus, strongly hydrogen-bonded hydroxyls give a ^1^H NMR signal in the lower-shielding region and are less mobile. It may be dangerous to interpret the other dominant peaks in the same way, because Si–O–H dynamics in a *np*-Si system might be much different from the dynamics on a silica surface.

A plausible identification of at least a major portion of the 6.0, 4.8 and 4.5 ppm spectral contributions is hydrogens that are *directly* bonded to silicon. This kind of hydrogen is essentially immobile, so the ^1^H–^1^H interactions between them are not averaged by atomic-level motion and are expected to be stronger than those between “mobile” hydroxyl protons of a comparable inter-hydrogen distance [45]. The ^29^Si CP–MAS spectra and related theoretical calculations of ^29^Si chemical shifts (*vide supra*) also indicate that a major fraction of the surface sites in *np*-Si samples that have not been intentionally oxidized consists mainly of hydrogen-terminated silicon, instead of silanol (Si–OH) groups (*vide infra*). This is consistent with our interpretation that these peaks in the DEPTH-echo measurements on *np*-Si for *τ*_total_ = 30 µs, arise mainly from hydrogen atoms that are *directly* bonded to surface silicon atoms.

*Water-Treated Samples.* Figure 3D,G show the proton-decoupled ^29^Si CP–MAS NMR spectra of *np*-Si that has been treated, respectively, with ^1^H_2_O or ^2^H_2_O (then evacuated at 150 °C), obtained using a 14 ms CP contact time. Comparison of these spectra shows the effect of a chemical treatment that provided an opportunity for replacement of any readily-exchangeable hydrogens on the surface (e.g., of Si–OH) by deuterium atoms. One sees no substantial evidence in Figure 3D or Table 1 of a removal of ^1^H sources for ^1^H– > ^29^Si CP. The proton-decoupled ^29^Si CP–MAS NMR spectrum of ^1^H_2_O-treated *np*-Si (Figure 3G) is very similar to the spectra of 150 °C-evacuated *np*-Si (Figure 3B) and the sample treated with ^2^H_2_O (Figure 3D). While there are some significant, albeit not dramatic, differences in the intensities of the six contributions (Table 1) for comparable positions in the spectra of Figure 3B,D and G, the line widths of comparable contributions are identical (within experimental error). The similarities among the ^29^Si CP–MAS spectra of Figure 3B,D and G are so strong that the only chemically significant conclusion that seems to follow is that *readily-exchangeable* *OH groups are not dominant on the surfaces of these samples;* otherwise, the effects of ^1^H–^2^H exchange would have been more dramatic in these ^1^H– > ^29^Si CP–MAS ^29^Si spectra.

The total NMR-determined proton concentrations of the samples of Figure 4A’ (as-received) and Figure 4B’ (pentane-treated-then-evacuated at 150 °C) are essentially the same as each other; also the total ^1^H concentrations of the samples of Figure 4D (^2^H_2_O-treated-then-evacuated at 150 °C) and Figure 4G (^1^H_2_O-treated-then-evacuated at 150 °C) are the same as each other. Within this quartet of samples/spectra, the pentane-treated and as-received samples have about 12% higher total ^1^H intensity than the samples that have been treated with water. This difference is primarily manifested in the intensity contributions around 3.9 ppm and 3.3 ppm and is partially compensated by larger contributions around 1.1 ppm for the water-treated samples. If there were a dominant contribution of readily exchangeable Si–OH moieties on the untreated (or pentane-treated-then-150 °C evacuated) sample, the main effect of ^2^H_2_O treatment would be hydrogen exchange and a reduced Si–O–^1^H intensity (probably at about 1.1 ppm) [44]; *such a spectral difference is not observed between* *Figures 4D,G, or between* Figure 4*D and* *Figure 4A*’ *or B*’. These results are consistent with indications from the ^29^Si NMR results (and ^2^H MAS results, *vide infra*) that there is not a dominant contribution of readily exchangeable Si–OH moieties on the un-oxidized *np*-Si surface.

An obvious difference between the ^1^H NMR spectra of ^2^H_2_O-treated and ^1^H_2_O-treated samples would have been expected, and is *not* observed, if water treatment had resulted in simple hydrolysis of strained Si–Si bonds, e.g., as represented by Equation 6.

*Si*–*Si* + H_2_O → H–*Si* + *Si*–OH (6)

A direct oxidation by water (Equation 7) seems unlikely.

*Si*–*Si* + H_2_O → *Si*–O–*Si* + H_2_ (7)

Table 2 indicates that the main ^1^H NMR effect of *np*-Si treatment with ^1^H_2_O (Figure 4G) and ^2^H_2_O (Figure 4D) is a transfer of ^1^H intensity from the region around 3.9 ppm (near the assignment for (*Si*–O–)_3_**Si**–H to about 1.1 ppm (near previous assignment for isolated silanols of a silica system) [44]. This decrease in 3.9 ppm intensity and increase in 1.1 ppm intensity could be explained by some kind of chemical reaction of the type shown in Equation 8 (assuming that the hydride ^1^H chemical shift of structure II is substantially different from that in structure I). 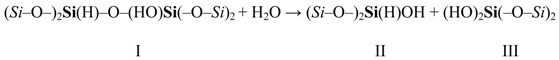
(8)

It may be noteworthy that the ^29^Si CP–MAS spectra (Figure 3D,G) of samples that correspond to those of the ^1^H MAS spectra of water-treated samples (Figure 4D,G) do not show any convincing evidence that supports (or refutes) the speculative chemistry embodied in Equations 6–8, or of *any* substantive chemical changes.

*np*-Si that had been treated with another hydroxyl-containing species, CH_3_OH, and then evacuated at 150 °C (Figure 5a), yielded a *lower* overall hydrogen concentration and a broader, reduced-intensity pattern around the central maximum, between about 3 ppm and about 5 ppm. This overall reduction of hydrogen concentration seems unlikely to be due to an extraction of non-polar organic contaminants, as these would be more effectively extracted by pentane; and it is not obvious from Figure 4 and Figure 5 that this occurs. One can speculate that the reduced-intensity pattern around 4 ppm might be due to some kind of chemical reaction involving the reduction of CH_3_OH (Equation 9); but these experiments showed no visual evidence (e.g., CH_4_ evolution) for such a process.

(*Si*–O–)_3_**Si**–H + CH_3_OH → (*Si*–O–)_3_**Si**–OH + CH_4_ (9)

*Oxidized samples*. Figure 3E shows the proton-decoupled ^29^Si CP–MAS NMR spectrum of oxidized *np*-Si (evacuated at 500 °C), obtained using a 14 ms CP contact time. The most obvious differences between the ^29^Si NMR spectra of oxidized (Figure 3E) and un-oxidized (Figure 3B) samples are a general loss of overall spectral intensity and peak narrowing in the spectrum of the oxidized sample and the total loss of intensity in the −14 ppm, −74 ppm and −83 ppm regions. One sees in Table 1 that only the spectral contributions centered at −89 ppm, −99 ppm and −109 ppm survive in the oxidation (the −109 ppm contribution may actually increase); as pointed out above, these are the same chemical shifts at which one finds the Q2, Q3 and Q4 moieties in the ^29^Si CP–MAS spectra of silicas [43]; hence, such assignments may account for *at least* portions of the spectral intensity in these chemical shift regions in the spectra of Figure 3. In terms of the tentative structural assignment given above, these results indicate that the 500 °C O_2_ treatment converts all **Si**–*Si* and **Si**–H bonds, and some of the **Si**–OH moieties, at the surface primarily to **Si**–O–*Si* linkages and that **Si** of most of these new **Si**–O–Si linkages are sufficiently remote from hydrogen atoms to be incapable of exhibiting observable ^1^H– > ^29^Si CP.

In the ^1^H MAS spectra, the three samples with a history of intentional oxidation (O_2_ treatment at 500 °C), represented in Figure 4E,F and Figure 5d, show dramatic changes relative to as-received (Figure 4A’) or pentane-treated-then-150 °C-evacuated (Figure 4B’) *np*-Si samples. The sample that was subjected to *only* 500 °C O_2_ treatment, with no subsequent water treatment (Figure 4E), shows a sharp decrease (roughly half) in total ^1^H concentration; Table 2 indicates that this intensity loss is distributed throughout the entire spectrum, except perhaps for the contribution at about 3.3 ppm. This broad intensity loss would be consistent with the occurrence of oxidation processes that convert **Si**–H moieties into **Si**–O–*Si* moieties or into **Si**–OH groups (Equation 10) followed by condensation of adjacent silanols (Equation 11).

2(*Si*–O–)_3−*n*_(*Si*–)*_n_***Si**–H + O_2_ → 2(*Si*–O–)_3−*n*_(*Si*–)*_n_***Si**OH (10)

2(*Si*–O–)_3−*n*_(*Si*–)*_n_***Si**OH → (*Si*–O–)_3−*n*_(*Si*–)*_n_***Si**–O–**Si**(–*Si*)*_n_*(–O–*Si*)_3−*n*_ + H_2_O (11)

The fact that, as seen in Table 2, the 1.1 ppm intensity in Figure 4A’,B’ (as-received or pentane-treated-then-150 °C evacuated, respectively) is largely retained in Figure 4E (500 °C oxidized) is consistent with the idea that dehydration is more difficult with isolated silanols (about 1.1 ppm) than with hydrogen-bonded silanols (>2 ppm). In light of the ^29^Si CP–MAS results (Figure 3) on the −109 ppm spectral contribution, It would appear that *n* = 0 may be the most probable interpretation.

Figure 3F shows the proton-decoupled ^29^Si CP–MAS NMR spectrum of oxidized-then-^1^H_2_O-treated (then evacuated at 150 °C) *np*-Si, obtained using a 14 ms CP contact time. This spectrum is very similar to that of the *np*-Si sample that had been only oxidized (no ^1^H_2_O treatment, Figure 3E), as shown numerically in Table 1. Thus, the ^29^Si CP–MAS experiments provide no obvious evidence of any major chemical transformation due to the water treatment.

The ^1^H MAS spectrum of the ^2^H_2_O-treated-then-oxidized sample (Figure 5d) shows a huge decrease in total ^1^H concentration, relative to that of the pentane-treated-then-150 °C evacuated *np*-Si sample, manifested across the entire spectrum, except for a small intensity increase in the 1–3 ppm range (Table 2). The large total ^1^H intensity loss upon oxidation of *np*-Si with O_2_ at 500 °C (Figure 4E) is more than restored when this sample is treated with ^1^H_2_O (Figure 4F); in fact the total ^1^H content of this sample is almost double that of the as-received or pentane-treated-then-150-°C-evacuated samples. From Table 2 one sees that this increase in total ^1^H content, relative to the spectrum of the O_2_-oxidized-only sample (Figure 4E), is spread across the spectrum, except for a modest decrease in spectral intensity around 3.3 ppm, and is especially large in the 4.8 ppm, 2.2 ppm and 1.1 ppm contributions. These intensity increases with ^1^H_2_O treatment are consistent with surface reactions of the types represented in Equations 12 and 13 for the oxidation and water-treatment steps and imply that the 4.8 ppm, 2.2 ppm and 1.1 ppm chemical shifts may be associated with structures of the (*Si*–O–)_4−*n−p*_ (*Si*–)*_p_***Si**(–OH)*_n_* type on the surface. For *p* = 0,

(*Si*–O–)_3−*n*_(*Si*–)*_n_***Si**–**Si**(–*Si*)*_m_*(–O–*Si*)_3−*m*_ + 1/2O_2_ → (*Si*–O–)_3−*n*_(*Si*–)*_n_***Si**–O–**Si**(–*Si*)*_m_*(–O–*Si*)_3−*m*_ (12)

(*Si*–O–)_3−n_(*Si*–)*_n_***Si**–O–**Si**(–*Si*)*_m_*(–O–*Si*)_3−*m*_ + H_2_O → (*Si*–O–)_3−*n*_(*Si*–)*_n_***Si**–OH + HO–**Si**(–*Si*)*_m_*(–O–*Si*)_3−*m*_ (13)

these proton chemical shifts have been assigned in previous work on silica gel to protons in isolated silanols (Si–OH) (1.1 ppm) [44], silanols with hydrogen-bonds (Si–OH^...^HO–Si) of modest strength (roughly 1.2 to 2.0 ppm) and silanol networks with very strong hydrogen bonds (>2 ppm). The modest decrease in 3.3 ppm intensity in the ^1^H MAS spectrum upon ^1^H_2_O treatment suggests that one effect of water treatment on the 500 °C/O_2_-oxidized *np*-Si sample could be a “sharpening” of the spectral intensity patterns on both sides of 3.3 ppm, ostensibly due to some degree of reduced structural heterogeneity in the associated site structures, reducing the overlapped intensity into the 3.3 ppm region.

Comparison of the spectra of oxidized samples that have been treated with ^1^H_2_O (Figure 4F) and with ^2^H_2_O (Figure 5d) emphasizes structural changes that are generated by oxidation, but show up in ^1^H NMR spectra of water-treated samples only when the water is ^1^H_2_O. This comparison shows larger spectral intensity contributions across the entire spectrum (Table 2) of Figure 4F, compared with Figure 5d, with the largest differences for the contributions at 4.8 ppm, 2.2 ppm and 1.1 ppm. Since one expects that the main source of differences between these ^1^H MAS spectra will be associated with readily “exchangeable” Si–OH moieties, these results indicate that these three spectral regions perhaps correspond to structural sites of the type, (*Si*–O–)_4−*n*_**Si**(–OH)*_n_* (most probably with *n* = 1 and 2). These chemical interpretations are consistent with the corresponding ^29^Si CP–MAS results, which show a dramatic loss of intensity centered at about −80 ppm, (*Si*–O–)_3_**Si**–H and (*Si*–O–)_2_**Si**(H)OH, for oxidized samples (Figure 3E for oxidized-only and Figure 3F for oxidized-then-^1^H_2_O-treated). The combination of results suggests that most likely there are essentially no Si–Si bonds remaining at the surface after O_2_ oxidation at 500 °C.

*^2^H MAS NMR results*. Since useful information regarding the chemical status of hydrogen atoms is often available from deuterium NMR, especially from comparing the NMR results of samples that differ only in their ^1^H *vs*. ^2^H contents, ^2^H MAS experiments were carried out on *np*-Si samples that had been (a) treated (exchanged) with ^2^H_2_O (the same kind of sample as in Figure 3D and Figure 4D) and (b) oxidized-then- ^2^H_2_O-treated (the same kind of sample as in Figure 5d). While most solid-sample ^2^H NMR experiments are carried out on static samples, focusing on the relationship between line shape and motion [53], our experiments employed MAS because of our primary interest in chemical shift/structure issues. Treating the *np*-Si with ^2^H_2_O will result in the replacement by ^2^H atoms of those ^1^H atoms that are both sterically accessible to the ^2^H_2_O and of a local site structure, e.g., Si–OH, that is suitable for exchanging hydrogen with liquid water (presumably via an adsorbed-water intermediate).

**Figure 8:**
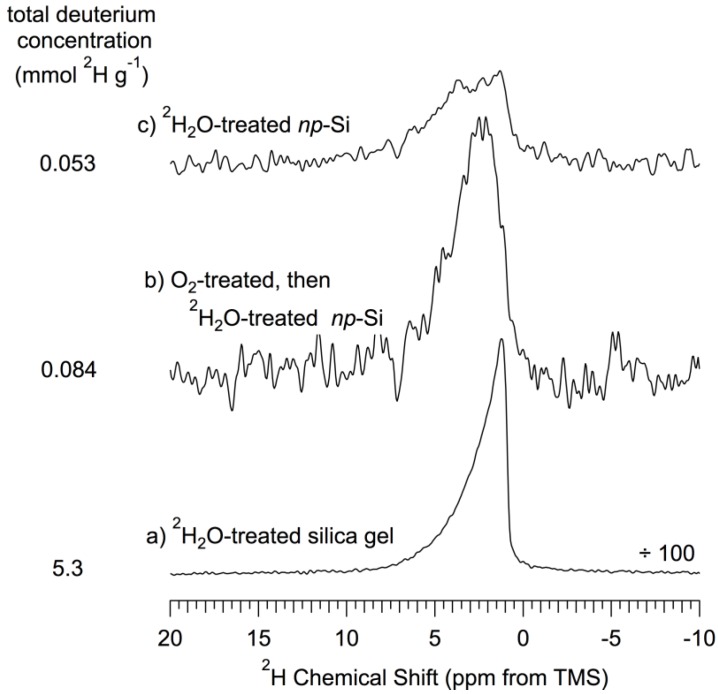
^2^H (55 MHz) MAS (7 kHz) NMR spectra of treated silica gel and treated *np*-Si samples. (**a**) ^2^H_2_O-treated-then-150 °C evacuated silica gel; (**b**) 500 °C O_2_-treated-then-^2^H_2_O-treated-then-150 °C evacuated *np*-Si; (**c**) ^2^H_2_O-treated-then-150 °C evacuated *np*-Si. The total deuterium concentration was determined by comparison of the numerical integral of each spectrum referenced to the proton concentration for dry silica gel determined by Li *et al.* [54]. The *np*-Si spectra are scaled so that the total area (integral) is proportional to the ^2^H g^−1^ value for each sample. The silica gel spectrum is scaled correspondingly, except for a factor of 100.

The results are shown in Figure 8, along with a ^2^H MAS spectrum of ^2^H_2_O-treated silica gel. This figure shows a ^2^H NMR signal that is nearly twice as intense for the oxidized-then-^2^H_2_O-treated sample (Figure 8b) as for the sample that had not been subjected to the O_2_/500 °C oxidation procedure (Figure 8c). Since the effect of ^2^H_2_O treatment is viewed substantially in terms of ^2^H-for-^1^H exchange in silanol groups at the surface, this result implies that the O_2_/500 °C treatment roughly doubles the H-exchange accessible silanol concentration on the *np*-Si surface. In comparison with the spectrum of a ^2^H_2_O-exchanged silica gel (Figure 8a), one sees that the H-exchangeable silanol concentrations represented in Figure 8 are very small compared with those of silica gel [54].

The OH concentration of dry silica gel has previously been determined (5.3 mmol OH g^−1^) [54] and apparently almost this entire hydrogen population is exchangeable with ^2^H_2_O; then, from the ^2^H NMR intensities of Figure 8 and the weights of the individual samples, the OH concentrations of *np*-Si and oxidized *np*-Si could be estimated as 0.053 and 0.084 mmol H g^−1^, respectively, assuming that all of the ^2^H_2_O-exchangeable hydrogen on *np*-Si-based samples are readily exchangeable silanol moieties. Comparing these two numbers with the hydrogen contents given in Table 2, one can conclude that about 3% of the hydrogen content of pentane-treated-then-150 °C evacuated *np*-Si is in H-exchangeable surface silanols and for the oxidized *np*-Si sample this percentage is about 6%.

*These numbers are an order of magnitude smaller than what one would estimate from the integrated ^29^Si CP–MAS NMR intensities summarized in Table 1, if we assumed that the intensities centered at about −89 ppm, −99 ppm, and −109 ppm were due entirely to silica-like, i.e., readily exchangeable sites.* These results reinforce the implied interpretation above that a substantial portion of the Si–OH moieties of *np*-Si are not accessible for hydrogen exchange.

## 4. Summary and Conclusions

The combination of CP*–*^29^Si and ^1^H chemical shift results and time-domain NMR data based on magnetic dipole-dipole interactions yields a self-consistent interpretation of the surface chemical structure of *np*-Si and various treated *np*-Si samples. This combination of NMR-derived information leads to a picture of the surface of as-received *np*-Si as consisting of a dominant contribution of a Si–H layer, with a small but significant contribution of surface silanols; these types of ‘surface sites’ are connected to a crystal-like silicon core by Si–O–Si bridges and Si–Si bonds. It has been reported that a hydrogen-terminated silicon surface is oxidized under ambient air at room temperature to form a SiO_2_-type layer [55]. Apparently, when a H–**Si**(–*Si*)_3_ surface is oxidized, the formation of H–**Si**(–*Si*)*_n_*(–O–*Si*)_3−*n*_ is energetically favored, compared to formation of a surface hydroxyl, e.g., H–O–**Si**(–*Si*)_3_, configuration [21,22,23,24,25,26,27]. Thus, the broad −80 ppm contribution that is dominant in the ^29^Si CP–MAS spectra of samples that have not been deliberately oxidized (500 °C/O_2_) is assigned to monohydride silicon atoms that are each bonded to three bridging oxygen atoms, (*Si*–O–)_3_**Si**–H (with intensity probably centered at about −85 ppm), and to the related (*Si*–O–)_2_Si(H)OH sites (with intensity centered at about −75 ppm); these assignments are consistent with published studies aimed at generating Si-H moieties on a silica surface [42] and supported by quantum mechanical calculations of ^29^Si chemical shifts. Such calculations also suggest that a ^29^Si CP–MAS spectral contribution at about −14 ppm in the spectrum of unmodified *np*-Si could be due to structures of the type (*Si*–O–)_2_**Si**(H)(–*Si*) and/or (*Si*–O–)**Si**(H)(–*Si*)_2_, but time-domain NMR results that reflect ^29^Si–^1^H dipolar interactions do not support such arguments, pointing instead to structures of the types, (HO–)*_n_***Si**(–*Si*)*_m_*(–O–*Si*)_4−*m−n*_. Comparing integrated intensities of the various contributions to the ^29^Si CP–MAS spectra of 150 °C evacuated *np*-Si (Table 1) and taking into account the arguments above for a small interfacial region, suggest the following qualitative order of site populations: (*Si*–O–)_3_**Si**–H > (*Si*–O–)_3_**Si**OH > (*Si*O–)_4−*m−n*_(*Si*)*_m_***Si**(–OH)*_n_* ≈ (*Si*–O–)_2_**Si**(H)OH ≈ interface sites > (*Si*–O–)_2_**Si**(–OH)_2_ > (*Si*–O–)_4_**Si**. A combination of ^1^H and ^2^H MAS experiments provides evidence for substantial contributions of silanol groups (some of which are not H-exchangeable), along with the dominant Si–H sites, on the surface of unmodified *np*-Si; the populations of these silanol sites are dramatically increased by deliberate oxidation (500 °C O_2_). Results from the combination of experiments involving treatment with O_2_ and/or H_2_O imply, contrary to published reports [55], that it is O_2_, rather than H_2_O, that is primarily responsible for the oxidation of *np*-Si.

In addition to the silicon species giving rise to the CP–MAS ^29^Si NMR spectra, there are silicon structures that give rise to the main DP–MAS peak at −73 ppm identified with crystal-like interior silicon structures and small populations of silicon sites that give rise to the as-yet unassigned peaks at −52 ppm and −118 ppm.

The reported experiments provide a self-consistent view of the generation or elimination of silanols under hydrolysis, oxidation and condensation reactions. Some of the speculative chemical processes that may be relevant in accounting for the NMR data involve transfer of the elements of H_2_. This fact is of interest in connection with the suggestion that silicon-based materials may hold promise in the search for efficient hydrogen storage.

## Supporting Materials

Contains additional details on (1) ^13^C NMR spin counting analysis; (2) the simulation/deconvolution of ^29^Si CP–MAS spectra; (3) the DEPTH-echo technique; (4) the simulation/deconvolution of ^1^H DEPTH-echo MAS spectra; (5) the simulation/deconvolution of ^29^Si CP–MAS spectra of 150 °C evacuated *np*-Si as a function of dipolar-dephasing time.
