# Supplementary material for: The Surface of Nanoparticle Silicon as Studied by Solid-State NMR
Source: Materials (Basel). 2012 Dec 20;6(1):18–46. doi: 10.3390/ma6010018 (PMC5452113; doi:10.3390/ma6010018)
Supplement: Supplementary file 1 [file materials-06-00018-s001.pdf]

Supplementary Information

1. ^{13}C Spin Counting

^{13}C (50 MHz) CP–MAS NMR spectra were obtained on as-received *np*-Si and, for comparison, a mixture of 1% (w/w) hexamethylbenzene (HMB) with silica gel. The spectra are shown in Figure S1. Although CP–MAS spectra obtained at only one CP contact time (14 ms) do not permit direct quantitation, one can estimate from the spectral integrations, taking sample weights into account, that as-received *np*-Si contains less than a 1% (w/w) carbon content.

Figure S1. 50 MHz ^{13}C CP–MAS (3.5 kHz) spectra of (**A**) *np*-Si and (**B**) 1% HMB mixed with silica gel. In both cases 15,000 transients were collected, using 1 s repetition delays and a separately determined background signal was subtracted. In both samples the same probe and rotor configuration was used.

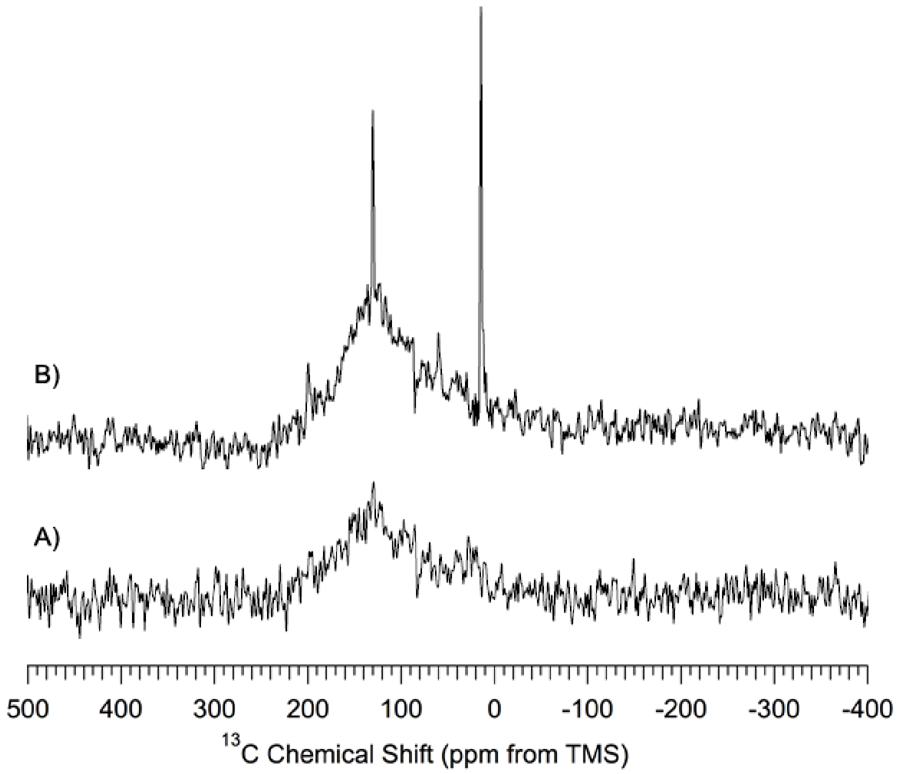

2. Deconvolution of ^{29}Si CP–MAS Spectra of Figure 3 (Summarized in Table 1)

Results shown here are presented in three parts—the experimental spectrum, the simulated spectrum based on the deconvolved spectral contributions and the difference between the first two. Figure S2 shows the experimental and simulated proton-decoupled ^{29}Si CP–MAS NMR spectra of *np*-Si evacuated at 150 °C. The experimental spectrum, as with all but that of Figure S3, was taken with a CP contact time (CT) of 14 ms.

Figure S2. Experimental and simulated proton-decoupled ^{29}Si (71.5 MHz) CP–MAS (7.0 kHz) NMR spectra of *np*-Si evacuated at 150 °C and their difference spectrum. A CP contact time of 14 ms was used.

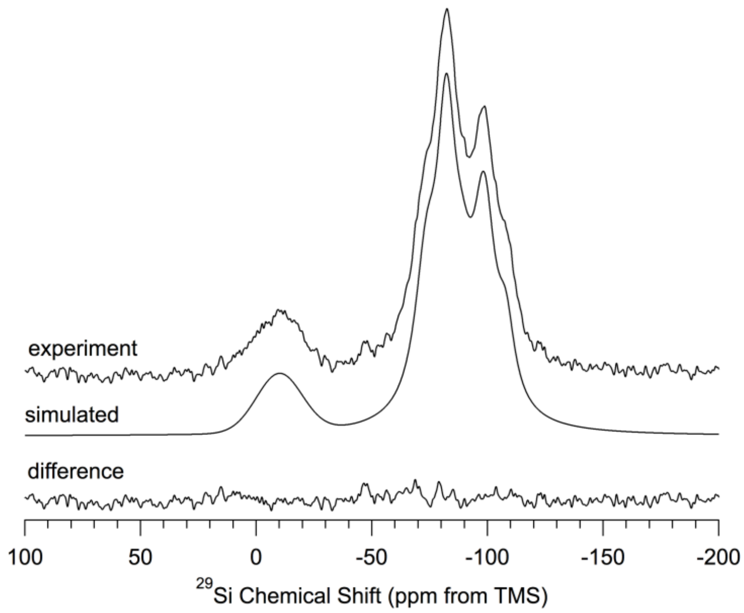

Figure S3. Experimental and simulated proton-decoupled ^{29}Si (71.5 MHz) CP–MAS (7.0 kHz) NMR spectra of *np*-Si evacuated at 150 °C and their difference spectrum. A CP contact time of 1.0 ms was used.

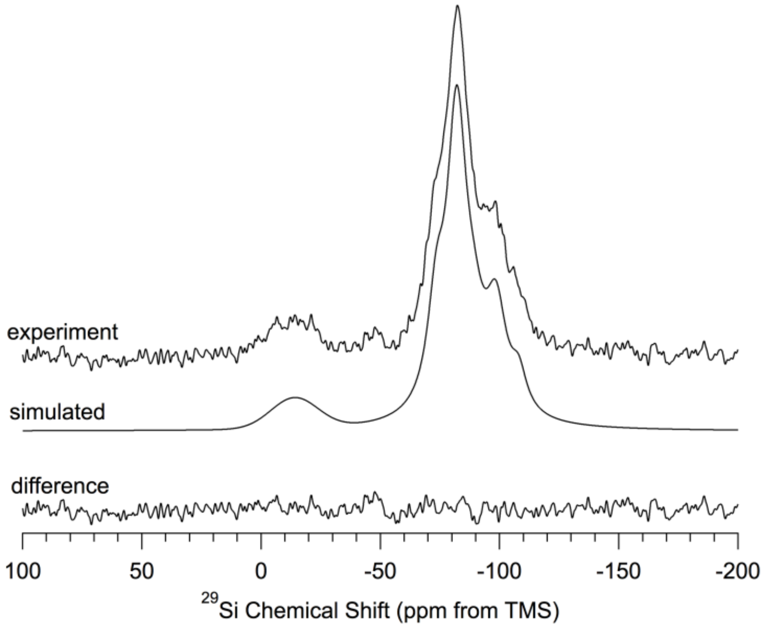

The simulated spectrum of Figure S2 consists of six heavily overlapping signals, each with a unique chemical shift, whose spectral characteristics are summarized, along with the other cases in this Section 2, in Table 1 and Table S1. One signal centered at −14 ppm has a Gaussian line shape, while the five others at −74, −84, −91, −100 and −109 ppm have Lorentzian line shapes. As with all of the spectra in this section, the use of a mixed (linear combination of Lorentzian and Gaussian) line shape for any of the signals did not improve perceptibly the match between the experimental and simulated

spectra. The signal at −14 ppm differs from the rest in having a significantly larger line width of 28 ppm, compared with 9 to 12 ppm for the others. No spinning sidebands are observed for any signal.

Figure S3 shows the experimental and simulated proton-decoupled ^{29}Si CP–MAS spectra of *np*-Si evacuated at 150 °C, taken with a 1.0 ms CP contact time. The line shape and line width of each signal in Figure S3 are very similar to the values of the corresponding signals in Figure S2, with the exception of the signal at −74 ppm, where the short CP contact time result is several ppm narrower than the long CP contact result. Although the difference is significant, it is small and its significance uncertain.

Figure S4 shows the experimental and simulated proton-coupled ^{29}Si CP–MAS NMR spectra of *np*-Si evacuated at 150 °C. The experimental spectrum was recorded without proton decoupling and using the same long CP contact time as used in Figure S2 (14 ms).

Figure S4. Experimental and simulated proton-coupled ^{29}Si (71.5 MHz) CP–MAS (7.0 kHz) NMR spectra of *np*-Si evacuated at 150 °C and their difference spectrum. A CP contact time of 14 ms was used.

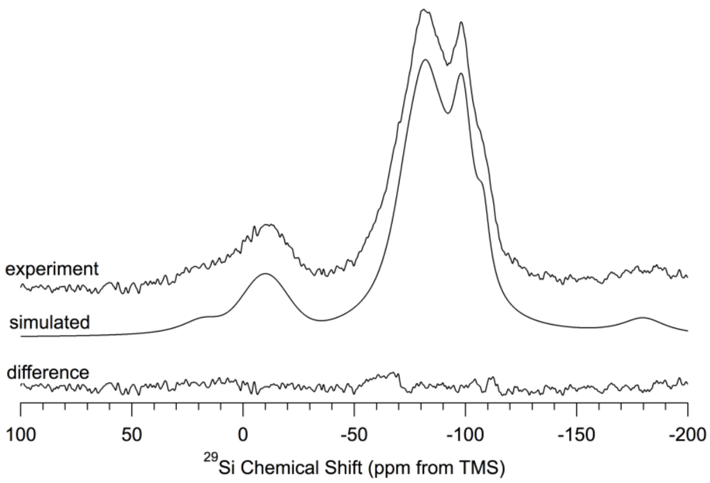

Figure S5 shows the experimental and simulated proton-decoupled ^{29}Si CP–MAS NMR spectra of $^{2}H_2O$-treated *np*-Si, obtained using a long CP contact time (14 ms). The simulated spectrum of Figure S5 consists of five heavily overlapping signals (Table 1). The signal at −14 ppm exhibits a Gaussian line shape. Signals at −74, −83, −89, −99 and −108 ppm have Lorentzian line shapes.

Figure S6 shows the experimental and simulated proton-decoupled ^{29}Si CP–MAS NMR spectra of oxidized *np*-Si, obtained using a long CP contact time (14 ms). The simulated spectrum of Figure S6 consists of three heavily overlapping signals (Table 1). The signals at −89, −99 and −109 ppm have Lorentzian line shapes. All of the other signals (−14, −74 and −83 ppm) seen in the spectra of the 150 °C evacuated samples are absent in the spectrum of this sample.

Figure S5. Experimental and simulated ^{29}Si (71.5 MHz) CP–MAS (7.0 kHz) proton decoupled NMR spectra of $^{2}H_2O$-treated *np*-Si and their difference spectrum. A CP contact time of 14 ms was used.

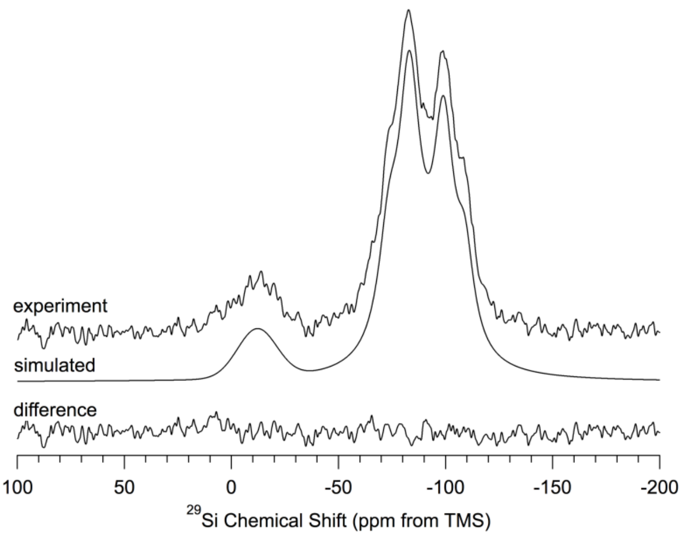

Figure S6. Experimental and simulated proton decoupled ^{29}Si (71.5 MHz) CP–MAS (7.0 kHz) NMR spectra of oxidized *np*-Si and their difference spectrum. A CP contact time of 14 ms was used.

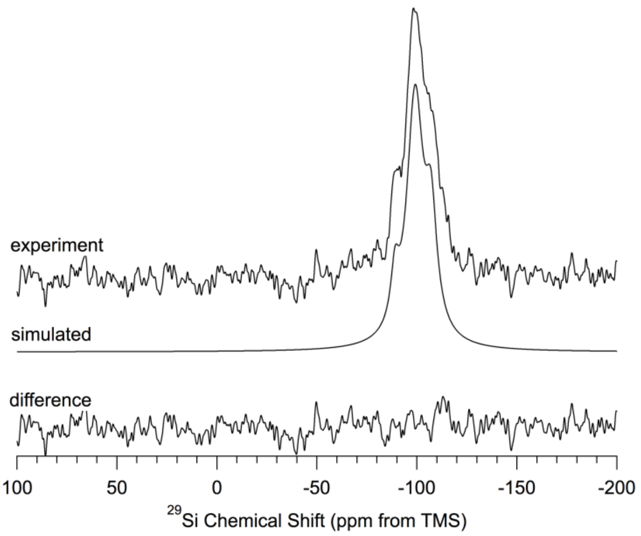

Figure S7 shows the experimental and simulated proton-decoupled ^{29}Si CP–MAS NMR spectra of oxidized-then-$^{1}H_2O$-treated *np*-Si, obtained using a long CP contact time (14 ms). The simulated spectrum of Figure S7 consists of three heavily overlapping signals (Table 1). Signals at −89, −99 and −109 ppm have Lorentzian line shapes.

Figure S8 shows the experimental and simulated proton-decoupled ^{29}Si CP–MAS NMR spectra of *np*-Si that has been treated with $^{1}H_2O$, obtained using a long CP contact time (14 ms). The simulated spectrum of Figure S8 consists of six heavily overlapping signals (Table 1). The signal at −14 ppm exhibits Gaussian line shape. Signals at −74, −83, −89, −99 and −109 ppm have Lorentzian line shapes.

Figure S7. Experimental and simulated proton-decoupled ^{29}Si (71.5 MHz) CP–MAS (7.0 kHz) NMR spectra of oxidized-then-$^{1}H_2O$-treated *np*-Si and their difference spectrum. A CP contact time of 14 ms was used.

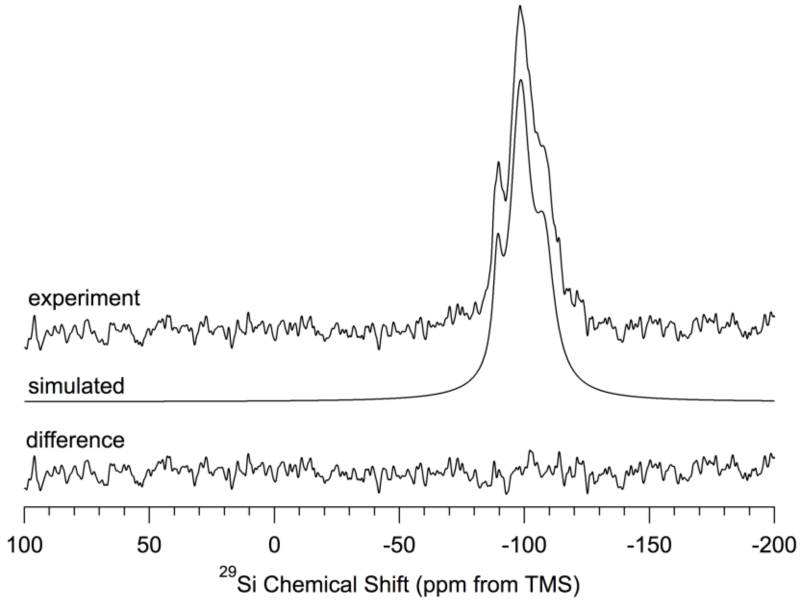

Figure S8. Experimental and simulated ^{29}Si (71.5 MHz) CP–MAS (7.0 kHz) proton decoupled NMR spectra of $^{1}H_2O$-treated *np*-Si and their difference spectrum. A CP contact time of 14 ms was used.

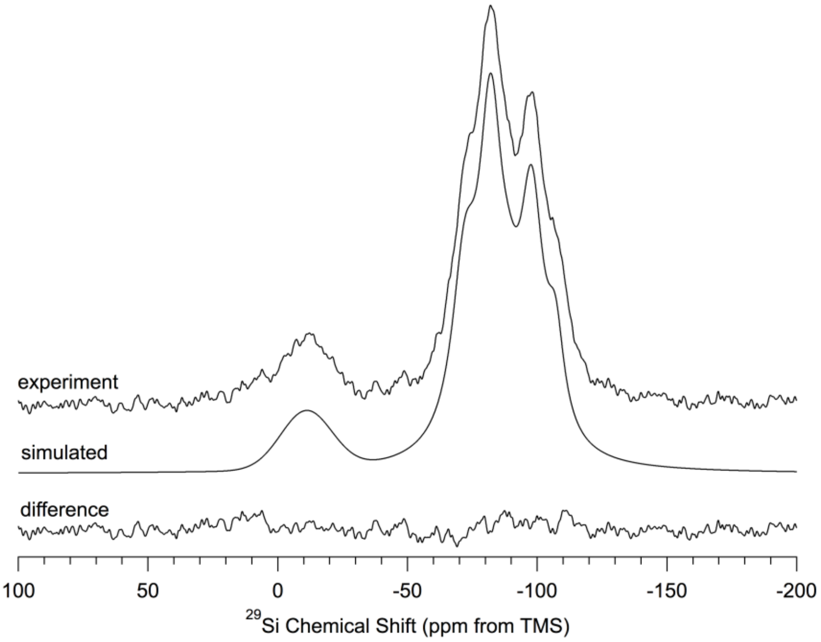

Figure S9 shows the experimental and simulated proton-decoupled ^{29}Si CP-MAS dipolar-dephased NMR spectra of *np*-Si evacuated at 150 °C.

Figure S9. Experimental and simulated proton-decoupled ^{29}Si (71.5 MHz) CP–MAS (5 kHz) dipolar-dephased NMR spectra of *np*-Si evacuated at 150 °C. A CP contact time of 14 ms was used. The values of dipolar-dephasing time used are shown on the figure.

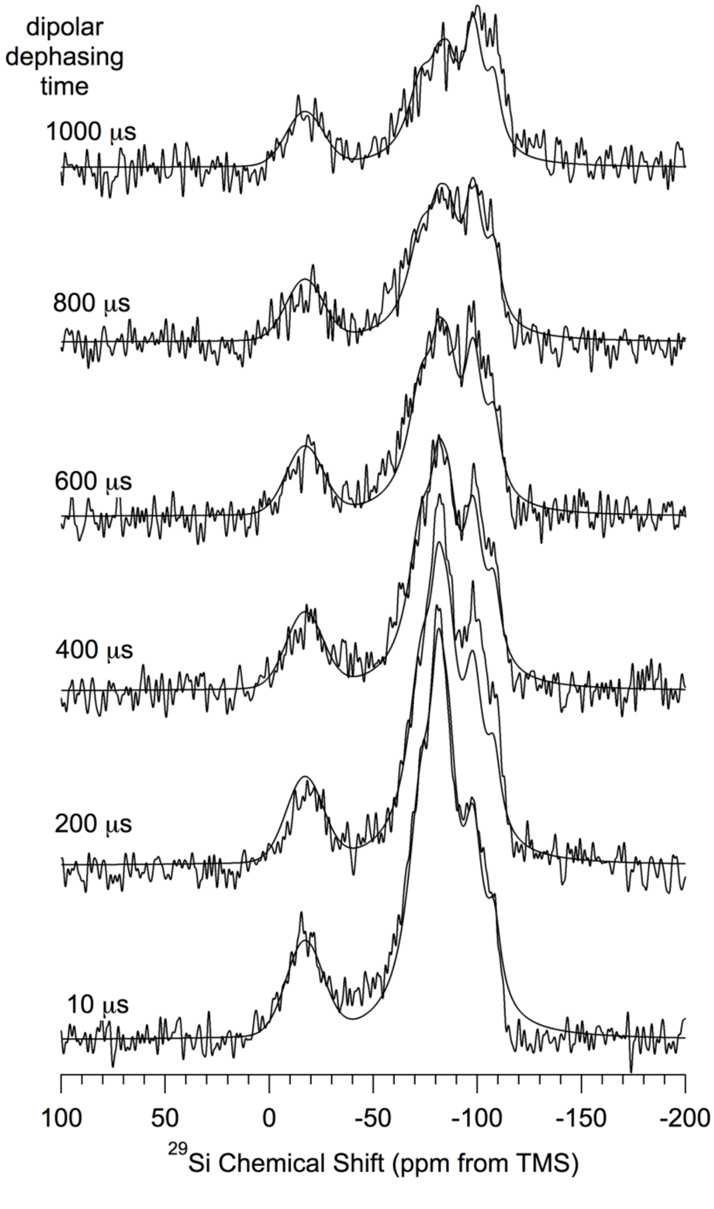

3. DEPTH-Echo Method

There are occasions when, in the course of measuring the NMR spectrum of a substance, especially one yielding relatively weak signals, the spectrum is contaminated by signals arising from the materials used to construct the NMR probe. Of course, NMR probes are generally constructed in such a fashion to avoid this spectral contamination but there are circumstances where it is unavoidable and the spectroscopist must adopt measures to eliminate it. Several strategies have been developed over the years to accomplish this decontamination; each has strengths and weaknesses, which makes it more or less applicable under specific circumstances.

One strategy is to record two NMR spectra, one with and one without the substance under study (the sample) in the probe (all other experimental variables are held constant) and to take the algebraic difference of these two spectra. This difference corresponds to the NMR spectrum of the sample free

of contamination. The process is often referred to as "background suppression through subtraction", where the term background refers, in particular, to the probe materials' signals and more generally to all signals other than those arising from the sample. It will be recognized that, if this prescription is followed exactly, the signal-to-noise ratio of the decontaminated spectrum suffers by a factor of $\sqrt{2}$ and that the total NMR instrument time is doubled over that needed to obtain the original NMR spectrum with the original S/N. The first drawback can be avoided by the introduction of an additional step in the process where the so-called "background spectrum", that is, the one without the sample, is replaced by a simulated version of it prepared by the spectroscopist and designed to match all of the observable spectral features but without the instrumental noise. The second drawback can be mitigated but perhaps not totally eliminated by characterizing the probe's background spectrum using a smaller amount of signal averaging or fixing upon the experimental conditions so that the background spectrum can be measured once and applied to many other measurements.

Other methods rely on the difference(s) between certain spectroscopic parameters of the substance under study and the probe materials, such as the longitudinal relaxation time, T_1, or the transverse relaxation time, T_2. Methods such as these can produce decontaminated spectra—provided there is a set of conditions where the spectroscopic distinction between the substance under study and the background signals is clear.

Another and powerful method, known as DEPTH [1,2], relies on a predominant RF design characteristic of NMR probes—the magnitude of the magnetic field rotating at the spectrometer's carrier frequency, $\mathbf{B_1}$, is *spatially inhomogeneous*. More precisely, the sequence capitalizes on the fact that there is a substantial difference between the magnitude of $\mathbf{B_1}$ at the sample and at background positions. This difference, together with the behavior of nuclear spins under the RF field-induced rotations, can be used to preserve the sample's signals and to suppress the background's signals (Figure S10).

Figure S10. Comparison of the structure of pulse sequences for Bloch Decay, DEPTH-1 (with one π pulse) and DEPTH-2 (with two π pulses). The pattern of RF phases in the rotating frame used to coherently average data varies among the pulse sequences.

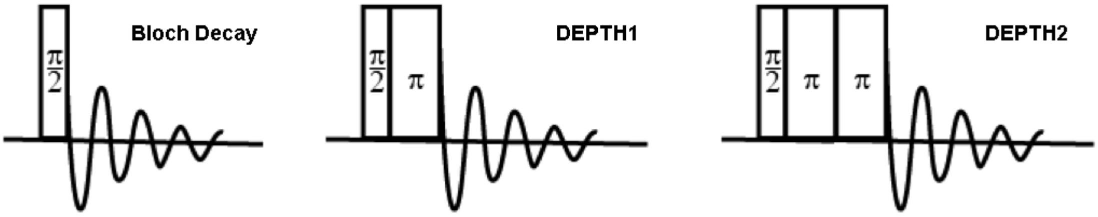

The principles of the mechanism of background suppression using DEPTH-style pulse sequences have been discussed extensively [1]. In the present work we deliberately include systematic variation of several free precession times in the DEPTH pulse sequences. See Figure S11. These varying times have been added for the purpose of suppressing the proton background signals of the NMR probe arising from the proton-containing materials used in its construction. These timing parameters also offer an additional degree of experimental flexibility to tease apart the heavily overlapping manifold of proton signals arising from the samples by taking advantage of differing T_2 values. A series of separate experiments using crystalline solid-state samples (data not presented) demonstrated that the

combination of DEPTH refocusing pulses and the inter-pulse times do not interfere with the suppression of background signals nor do they perturb the values of T_2 relaxation times measured on these samples by traditional methods.

Figure S11. Comparison of the structure of pulse sequences for chemical shift echo, DEPTH (with two π pulses) and DEPTH-echo (with two π pulses). The patterns of RF phases in the rotating frame for DEPTH2 and DEPTH-echo are unaffected by the inter-pulse spacing and are identical for the two sequences.

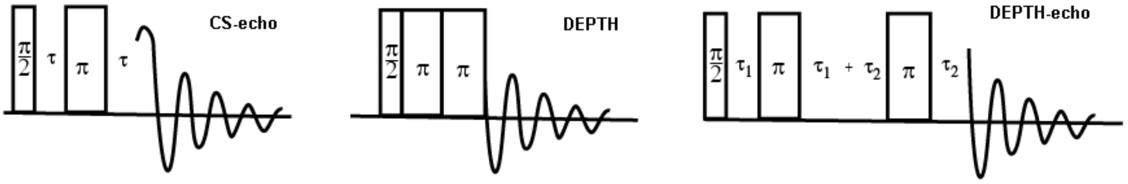

4. Simulation/Deconvolution of DEPTH-Echo ^{1}H MAS Spectra

^{1}H MAS spectra, obtained via the DEPTH-echo method with various values of $\tau_{total} = 2(\tau_1 + \tau_2)$ for the samples of Figures 4 and 5 were deconvolved/simulated in terms of nine possible spectral contributions. The simulations are shown in the Section 4 figures (Figures S12–S20) that follow. The plots for $\tau_{total} = 30$ μs correspond to Figures 4 and 5.

For each experiment, the experimental (noisy lines) and corresponding simulated spectra (smooth lines) for each value of τ_{total} time are overlapped to permit comparison. The derived intensities and widths of the spectral contributions are summarized in Table 2 and Table S2.

All signals exhibit Lorentzian line shapes. As with all of the spectra in this section, the use of a mixed (linear combination of Lorentzian and Gaussian) line shape for any of the signals did not improve the match between the experimental and simulated spectra.

Figure S12. Experimental (with noise) and simulated (without noise) ^{1}H (600.1 MHz) DEPTH-echo MAS (20 kHz) NMR spectra of *np*-Si evacuated at 150 °C. Various values of τ_{total} as noted in the figure were used in obtaining the experimental spectra.

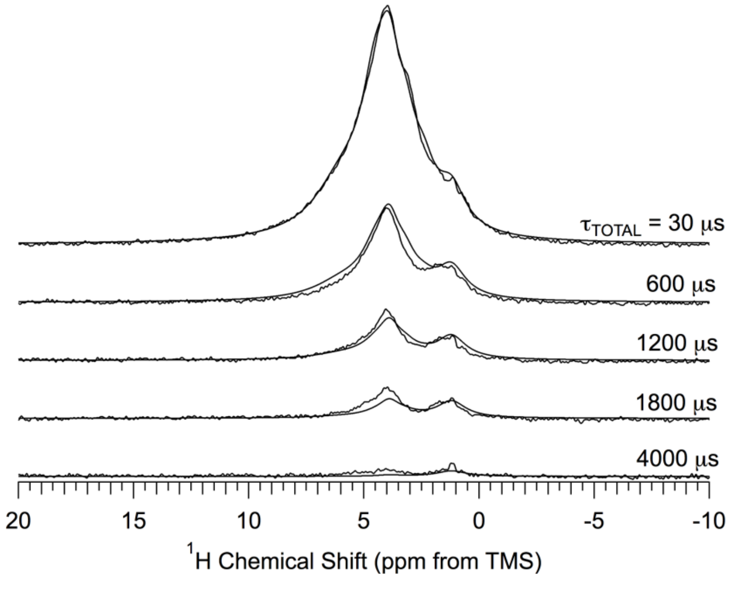

Figure S13. Experimental (with noise) and simulated (without noise) ^{1}H (360.1 MHz) DEPTH-echo MAS (16 kHz) NMR spectra of *np*-Si treated with $^{1}H_2O$-then-evacuated at 150 °C. Various values of τ_{total} as noted in the figure were used in obtaining the experimental spectra.

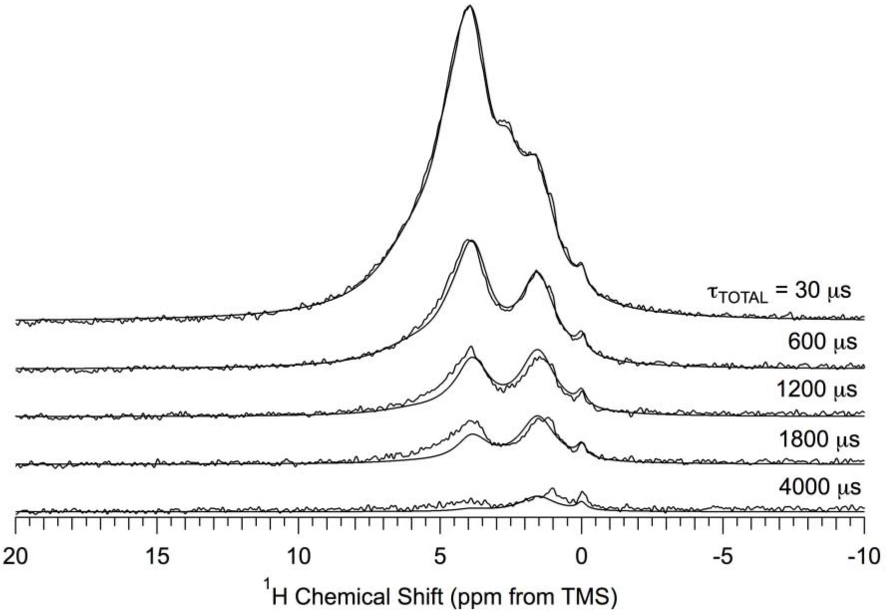

Figure S14. Experimental (with noise) and simulated (without noise) ^{1}H (360.1 MHz) DEPTH-echo MAS (16 kHz) NMR spectra of *np*-Si treated with $^{2}H_2O$-then-evacuated at 150 °C. Various values of τ_{total} as noted in the figure were used in obtaining the experimental spectra.

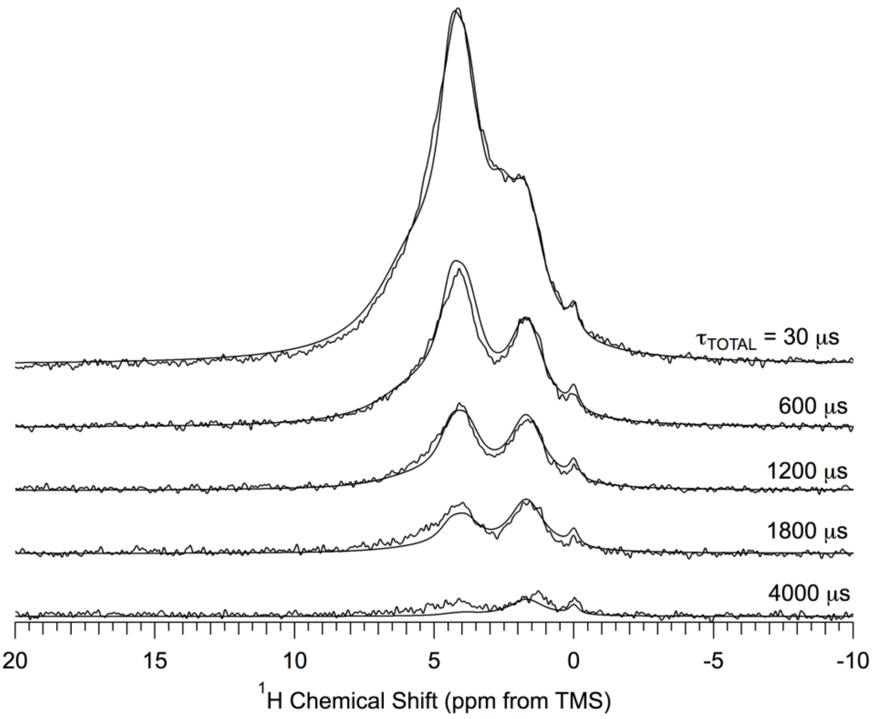

Figure S15. Experimental (with noise) and simulated (without noise) ^{1}H (360.1 MHz) DEPTH-echo MAS (15 kHz) NMR spectra of *np*-Si treated with CH_3OH-then-evacuated at 150 °C. Various values of τ_{total} as noted in the figure were used in obtaining the experimental spectra.

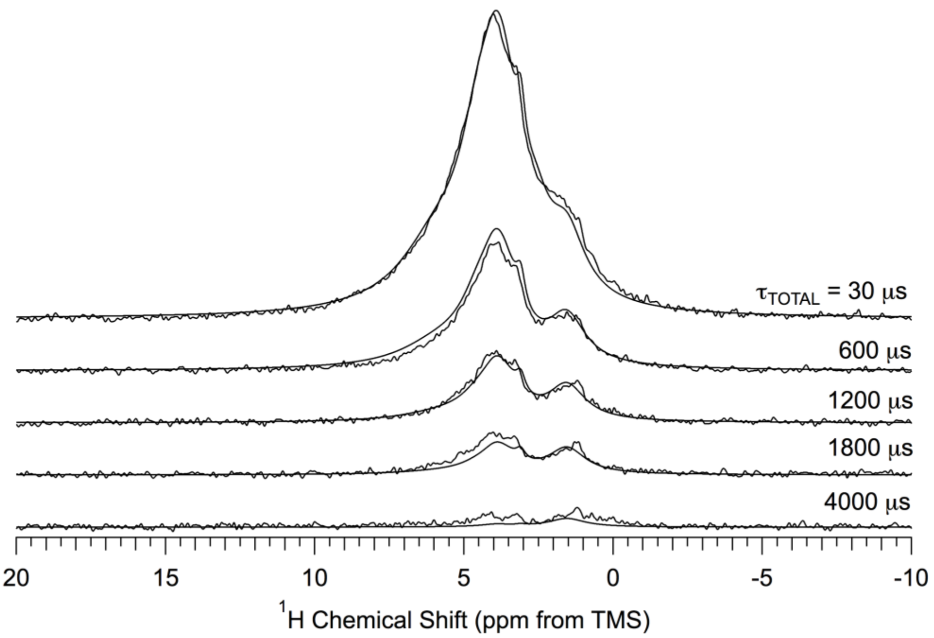

Figure S16. Experimental (with noise) and simulated (without noise) ^{1}H (360.1 MHz) DEPTH-echo MAS (15 kHz) NMR spectra of *np*-Si treated with pentane-then-evacuated at 500 °C. Various values of τ_{total} as noted in the figure were used in obtaining the experimental spectra.

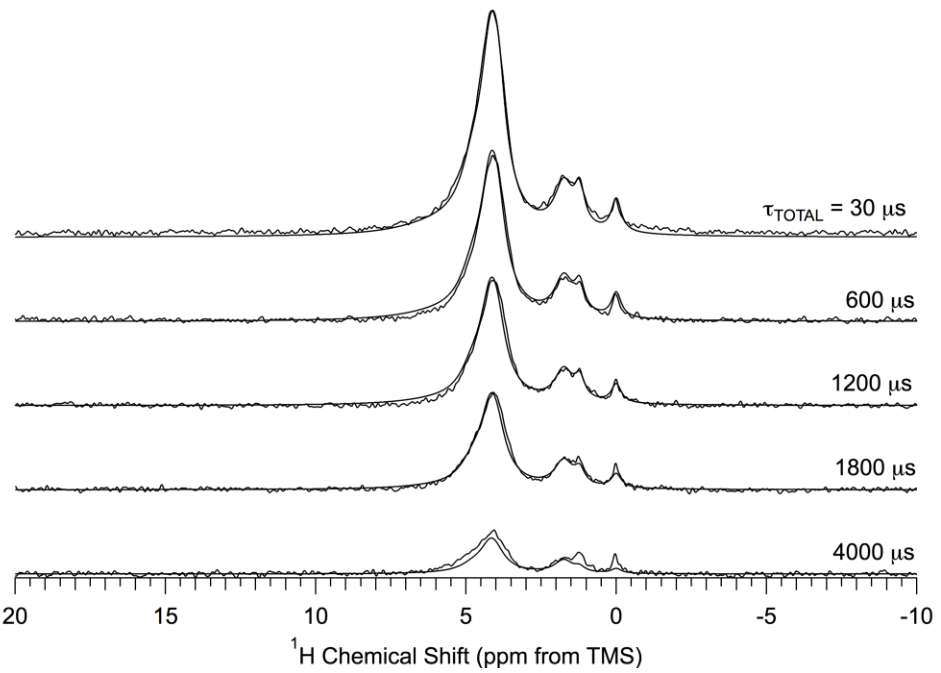

Figure S17. Experimental (with noise) and simulated (without noise) ^{1}H (360.1 MHz) DEPTH-echo MAS (15 kHz) NMR spectra of *np*-Si evacuated at 500 °C. Various values of τ_{total} as noted in the figure were used in obtaining the experimental spectra.

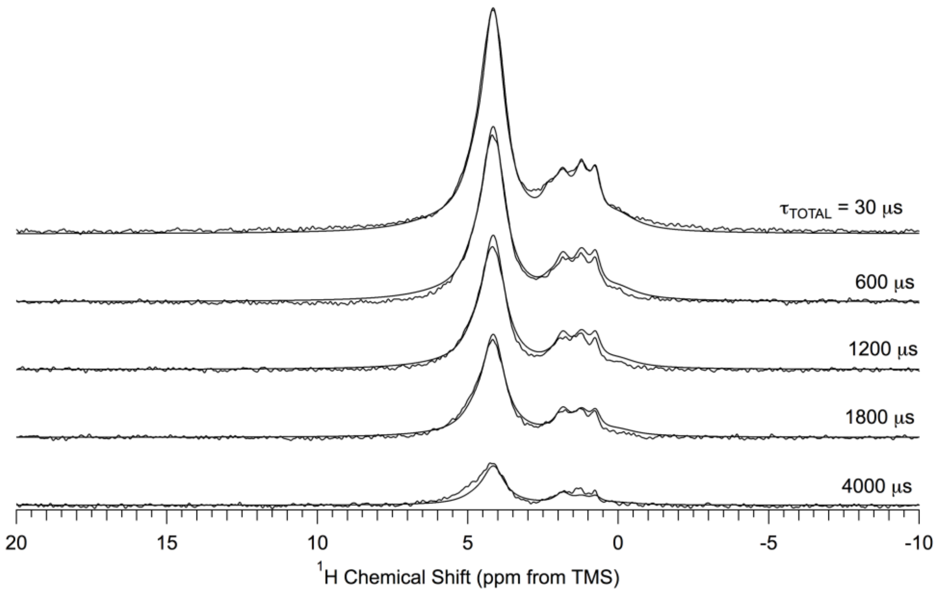

Figure S18. Experimental (with noise) and simulated (without noise) ^{1}H (360.1 MHz) DEPTH-echo MAS (15 kHz) NMR spectra of *np*-Si treated with oxidized at 500 °C-then-treated with $^{1}H_2O$-then-evacuated at 150 °C. Various values of τ_{total} as noted in the figure were used in obtaining the experimental spectra.

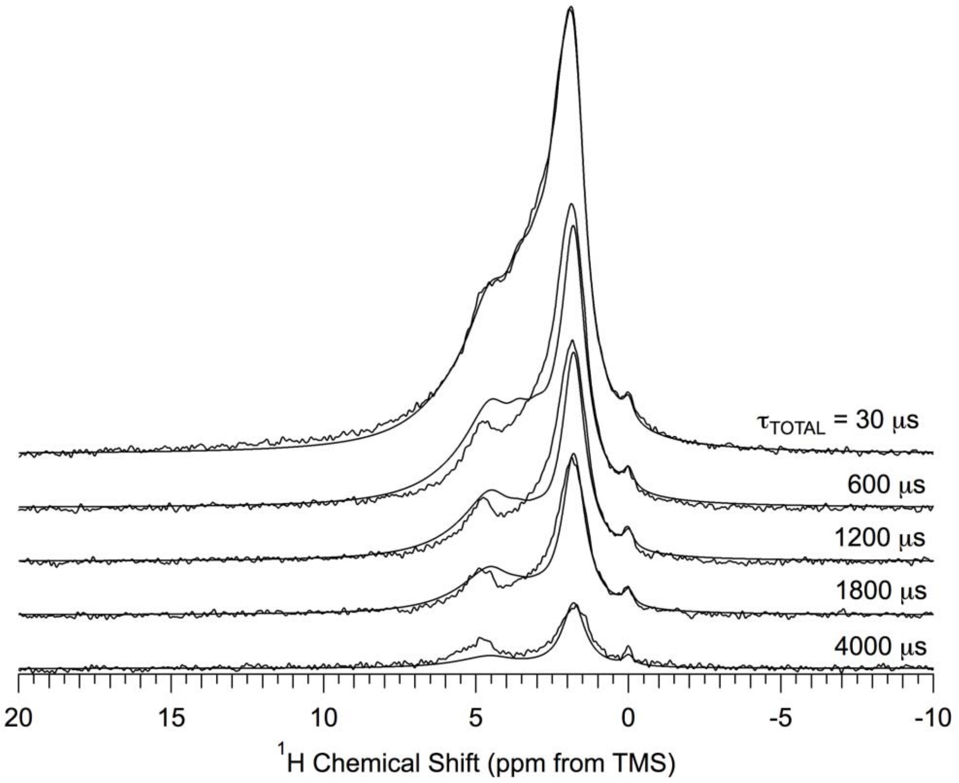

Figure S19. Experimental (with noise) and simulated (without noise) ^{1}H (360.1 MHz) DEPTH-echo MAS (17 kHz) NMR spectra of *np*-Si oxidized at 500 °C-then-treated with ^2H_2O-then-evacuated at 150° C. Various values of τ_{total} as noted in the figure were used in obtaining the experimental spectra.

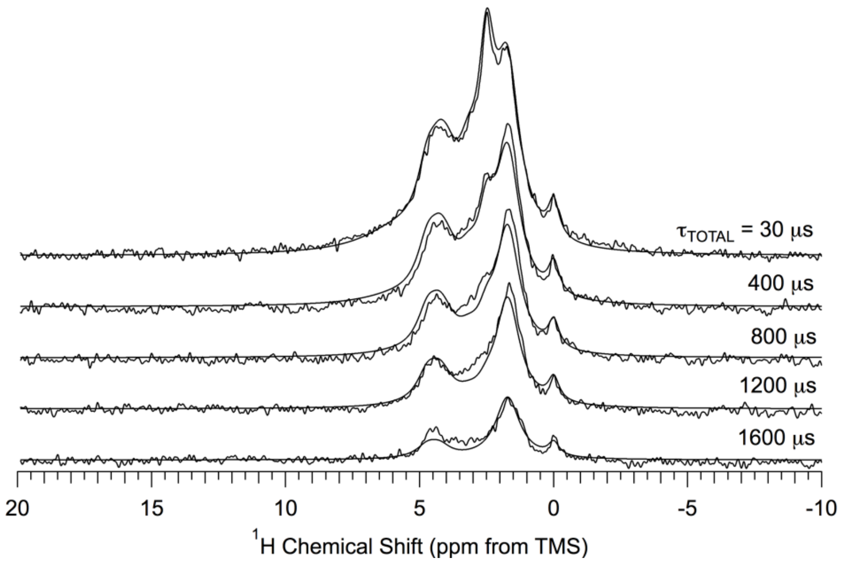

Figure S20. Experimental (with noise) and simulated (without noise) ^{1}H (360.1 MHz) DEPTH-echo MAS (17 kHz) NMR spectra of *np*-Si oxidized at 500 °C. Various values of τ_{total} as noted in the figure were used in obtaining the experimental spectra.

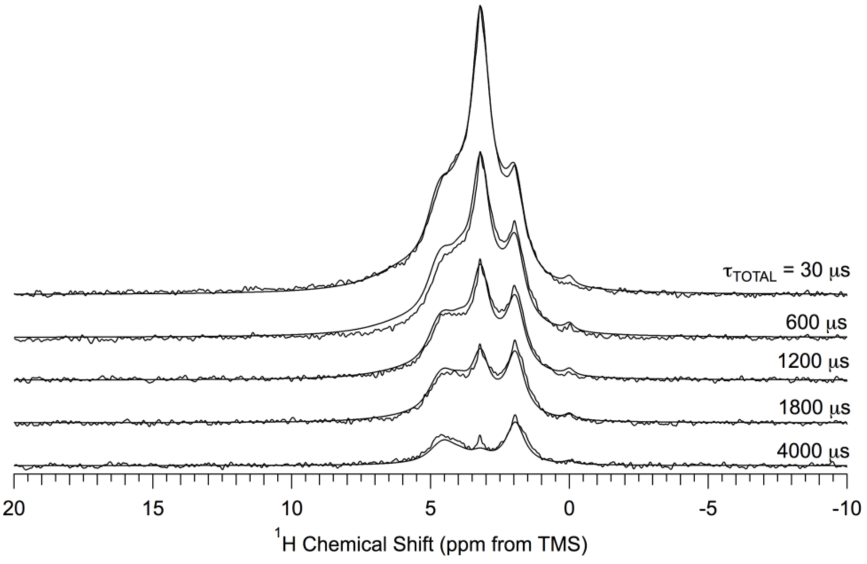

5. Simulation/Deconvolution of ^{29}Si CP–MAS Dipolar-Dephasing Results

Figure S21 contains experimental and simulated dipolar-dephasing ^{29}Si NMR CP–MAS spectra of *np*-Si samples as described in the main text. In the figure, the experimental (noisy lines) and corresponding simulated (smooth lines) spectra for each value of dipolar dephasing time are overlapped to permit comparison between them.

One signal centered at −14 ppm has a Gaussian line shape, while the five others at −74, −84, −91, −100 and −109 ppm have Lorentzian line shapes. As with all of the spectra in this section, the use of a

mixed (linear combination of Lorentzian and Gaussian) line shape for any of the signals did not improve the match between the experimental and simulated spectra.

Figure S21. Experimental and simulated ^{29}Si (71.5 MHz) CP–MAS (5 kHz) dipolar-dephasing NMR spectra of *np*-Si evacuated at 150 °C. Various values of interrupt interval, *τ*, as noted in the figure were used in obtaining the experimental spectra.

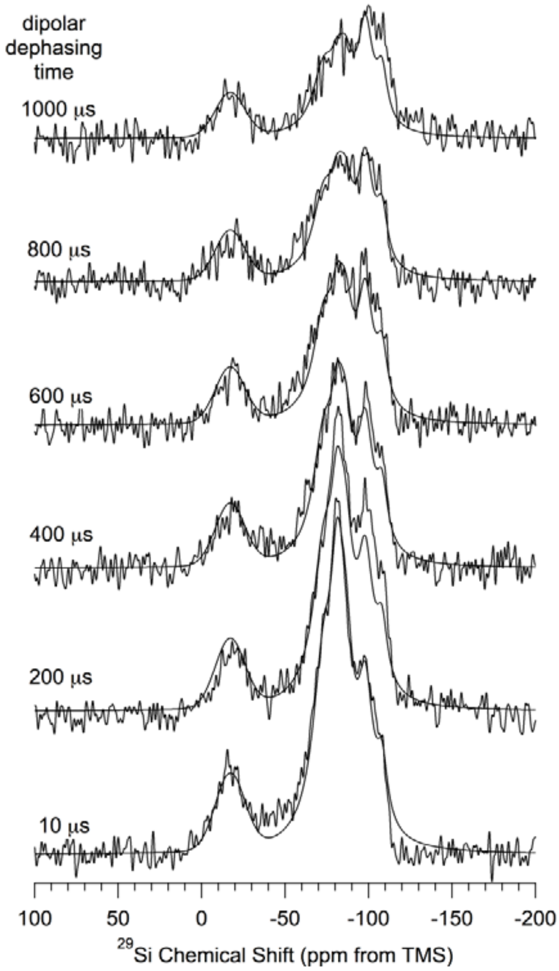

6. Tables

Table S1. Linewidths used in the simulations of ^{29}Si CP–MAS spectra of Figure 3 [a].

	Linewidth [b]						
**Sample/^{29}Si Chemical Shift (ppm)**	−14	−74	−83	−89	−99	−109	Total
1 ms CT (Figure 3A)	28	9	10	11	11	8	540
14 ms CT (Figure 3B)	28	12	10	11	12	9	1000
^{1}H coupled (Figure 3C)	28	24	18	12	12	7	1004
$^{2}H_2O$ treated (Figure 3D)	26	12	12	12	12	10	1130
500 °C O_2 (Figure 3E)	0	0	0	5	10	7	280
500 °C O_2, then $^{1}H_2O$ treated (Figure 3F)	0	0	0	4	10	9	310
$^{1}H_2O$ treated (Figure 3G)	28	12	11	10	11	8	1080

[a] Unless otherwise specified, all spectra were obtained with a CP contact time (CT) of 14 ms; [b] Estimated uncertainties: about ±10% of each reported value.

Table S2. Summary of the Signal Line Widths (FWHM) [a] of the Simulation/Deconvolution of ^{1}H MAS Spectra of Figures 4 and 5.

	Linewidth								
**Sample/ ^{1}H Chemical Shift (ppm)**	6.0	4.8	4.5	3.9	3.3	3.1	2.2	1.1	0.0
CH_3OH (Figure 5 [a])	3.0	3.0	1.5	1.1	1.2	0.4	1.2	1.5	0.4
Oxidized, then $^{2}H_2O$ (Figure 5d)	3.0	1.0	0.9	0.9	1.0	–	0.8	1.1	0.5
Oxidized, then $^{1}H_2O$ (Figure 4F)	3.0	2.8	1.5	1.0	1.0	–	1.0	0.9	0.4
$^{2}H_2O$ (Figure 4D)	3.0	3.0	1.0	1.0	1.2	–	1.2	1.5	0.4
$^{1}H_2O$ (Figure 4G)	3.0	3.0	1.5	1.1	1.2	–	1.2	1.5	0.4
Pentane, then 500 °C evacuated (Figure 5b)	3.0	1.0	0.9	0.5	–	–	–	0.6	0.4
500 °C evacuated (Figure 5c)	3.0	1.0	0.9	–	–	–	0.6	1.3	1.5
500 °C oxidized (Figure 4E)	3.0	1.0	1.0	1.0	0.8	–	1.0	0.8	0.4
Pentane, then 150 °C evacuated (Figure 4B')	3.0	3.0	1.5	1.1	1.2	–	1.2	1.5	0.4
as-received (Figure 4A')	3.0	3.0	2.0	1.8	1.8	2.0	0.4	0.4	0.4

[a] Estimated uncertainties: about ±10% of each reported line width.

References

1. Bendall, M.R.; Gordon, R.E. Depth and refocusing pulses designed for multipulse NMR with surface coils. *J. Magn. Reson.* **1983**, *53*, 365–385.
2. Cory, D.G.; Ritchey, W.M. Suppression of signals from the probe in bloch decay spectra. *J. Magn. Reson.* **1988**, *80*, 128–132.

© 2013 by the authors; licensee MDPI, Basel, Switzerland. This article is an open access article distributed under the terms and conditions of the Creative Commons Attribution license (http://creativecommons.org/licenses/by/3.0/).
